# Application of Hydrogels and Hydrocarbon-Based Gels in Oil Production Processes and Well Drilling

**DOI:** 10.3390/gels9080609

**Published:** 2023-07-28

**Authors:** Aleksey Telin, Lyubov Lenchenkova, Ravil Yakubov, Kira Poteshkina, Polina Krisanova, Andrey Filatov, Aleksandr Stefantsev

**Affiliations:** 1 Ufa Scientific and Technical Center, LLC, 99/3, Kirova Street, 450078 Ufa, Russia; 2 Faculty of Mining and Petroleum, Ufa State Petroleum Technological University, 1, Kosmonavtov Street, 450064 Ufa, Russia; lenchenkoval@mail.ru (L.L.); rnyakubov@gmail.com (R.Y.); 3 World-Class Research Center «Efficient Development of the Global Liquid Hydrocarbon Reserves», Faculty of Chemical and Environmental Engineering, National University of Oil and Gas «Gubkin University», 65 Lenin Avenue, Building 1, 119991 Moscow, Russia; poteshkina.k@gubkin.ru (K.P.); krisanova_polina@mail.ru (P.K.); filatovandrew104@gmail.com (A.F.); stefantsev.aa@gmail.com (A.S.)

**Keywords:** gel, hydrogel, hydrocarbon-based gel, structural and mechanical properties, filtration properties, hydraulic fracturing, conformance control, oil production stimulation, enhanced oil recovery, well drilling, well killing

## Abstract

The use of gels in oil production processes has become a regular practice in oilfield operations and is constantly developing in all oil-producing countries of the world, as evidenced by the growth of publications and patent activity on this topic. Many oil production processes, such as hydraulic fracturing, conformance control, water, and gas shutoff, cannot be imagined without the use of gel technologies. Inorganic, organic, and hybrid gels are used, as well as foams, gel-forming, and gel-dispersed systems. The possibility of a broad control of structural and mechanical properties, thermal stability, and shear resistance by introducing microscale and nanoscale additives made hydrogels and hydrocarbon-based gels indispensable tools for oil engineers.

## 1. Introduction

The use of hydrogels and hydrocarbon gels in oil production processes is very diverse and significantly affects many stages of the oil production process chain, such as well drilling, oil production stimulation, water and gas shutoff, injection well conformance control, and enhanced oil recovery. Organic and mineral gels are used, as well as gels made from hybrid organo–inorganic materials.

Partially hydrolyzed polyacrylamide [1] and guar polymers [2] have become the most commonly used materials of the water-soluble polymers that form the basis of gels, as well as silicon- [3] and aluminum-containing [4] compounds from inorganic materials. This direction is constantly developing. Oilfield service companies around the world are continuously improving their formulations both to increase process efficiency and reduce costs. Many extremely important operations, including hydraulic fracturing, conformance control, and gas and water shutoff in wells, are generally unthinkable today without the application of a variety of gels.

Guar and hydroxypropyl guar gels have become the most widespread, while xanthan and polyacrylamide gels are less commonly used in hydraulic fracturing [2,5,6,7,8,9,10]. Hydrocarbon-based gels are used for this as well [11,12,13]. Recently, gels based on viscoelastic surfactants have been used [14,15,16,17,18,19,20,21,22]. They have one clear advantage over other polymer gels: they are destroyed during well inflow after fracturing by both oil and water.

Water shutoff operations using hydrogels to reduce the idle circulation of injected water, intensify oil production, and enhance oil recovery are conducted both in injection (flow-diversion technologies) and production (remedial cementing) wells [23]. The qualitative composition of hydrogels used for both flow diversion and water shutoff is almost identical. Gels differ mainly in their concentration parameters: lower concentrations of primary components are used for flow diversion, and the structural and mechanical properties of hydrogels are weaker. For water shutoff, by contrast, the concentration of the primary components is many times higher, and such rheological characteristics of hydrogels as the ultimate shear strain, viscosity, and storage modulus are significantly higher. Moreover, depending on the type of remedial cementing operations (selective water shutoff, isolation of watered out formation intervals, elimination of behind-the-casing, casing leak repairs, etc.), hydrogels are used both in pure form and are combined with more rigid grouting materials, which are reinforced with curing resins or micro cement [24].

The use of hydrogels in well drilling is currently limited to the drilling of horizontal sections in clay reservoirs, but they are more often used for controlling disastrous lost circulation since viscoelastic gels reinforced with dispersed particles can effectively eliminate absorption zones [25].

Hydrogels are used as acid diverters for well stimulation by acid treatment. Various application options are possible, from polyacrylamide gels that have already become a conventional option to gels based on viscoelastic surfactants.

The purpose of this review is to identify trends in the development of chemistry and technology of gels when they are used in the oil industry, as well as to identify the most promising areas of scientific research in this area.

**Figure 1:**
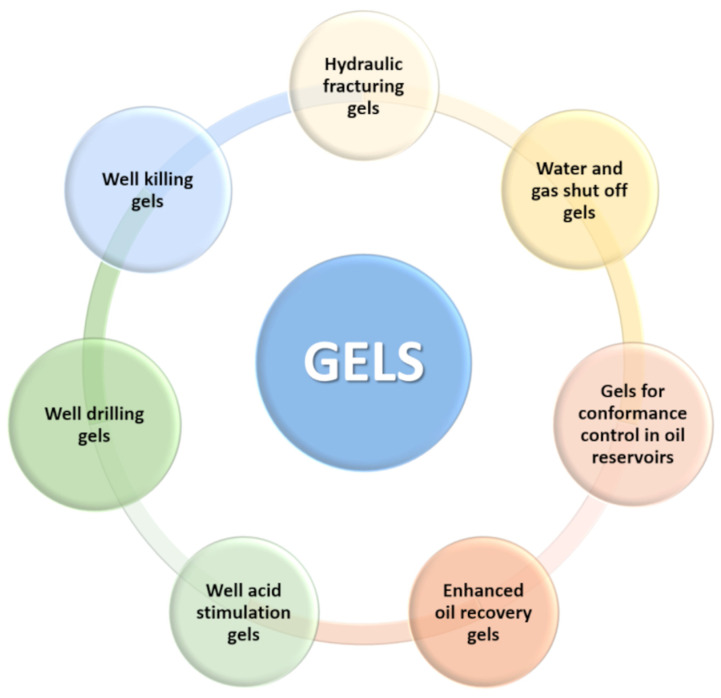
Oil industry processes in which gels are used.

Figure 1 shows a schematic illustration of various processes in the oil industry in which gels are used.

Gels can be classified according to various characteristics. In this review, the gels used in the oil industry are divided according to their application. Table 1 shows the compositions of the dispersion and dispersed phases of gels.

## 2. Hydraulic Fracturing Gels

Hydraulic fracturing is a well-known method for oil and gas production stimulation. The essence of the process is based on the injection of a composite viscous liquid or gel under pressure exceeding the pressure of fracturing. As a result, a system of fractures is formed, the length of which can reach several hundred meters. The permeability of fractures, as a rule, is several times higher than the initial permeability of the pores of collectors, which justifies the increased influx of fluids.

In most cases, a propping material is used in hydraulic fracturing such as a proppant or sand to prop the resulting fractures; that is, to prevent them from closing under the impact of rock pressure. Fracturing fluids should have stable rheological properties. Thus, during the entire duration of hydraulic fracturing, the viscosity of the gels should be the maximum for retaining the proppant in the volume, for its uniform distribution over the volume of fractures, and for the development of fractures of the required geometry. After the process, the viscous liquid is an obstacle to the filtration of reservoir fluids. Therefore, the viscosity of hydraulic fracturing gels decreases, i.e., the gels are destructed under the action of special destructor reagents or in contact with reservoir fluids.

The existing variety of hydraulic fracturing gels can be divided into two large classes: water gels (hydrogels) and hydrocarbon gels. Various modifications of compositions in the form of emulsion and foam compositions are already possible due to their base. There are also alcohol-based gels.

The development and successful application of technologies and various compositions for hydraulic fracturing have a history of almost 80 years, starting from the late 1940s. Only hydrocarbon-based gels were used at the first stages of technology development. Systems based on light fractions of hydrocarbons thickened with aluminum soap were used. Due to the high hazard of such compositions, they were later replaced by gels based on heavier fractions and emulsions. Their use did not damage the formation rock and formation fluids; they did not cause any swelling of clay particles with subsequent migration and clogging of pores and formed fractures; and they did not form emulsions and sediments with in-place oil.

Water gels for hydraulic fracturing were not used until the 1960s to avoid these problems. However, their studies continued, as they are cheaper, more environmentally friendly, and significantly less demanding on safety issues. Researchers showed that the introduction of potassium and calcium chlorides into the composition of the hydrogel can significantly reduce the damaging effect on water-sensitive formations. These studies opened up the possibility of using hydrogels in hydraulic fracturing. Since the 1970s, the possibility of using synthetic polymers as thickeners of aqueous media has been investigated. These are mainly polyacrylamide and its derivatives. Extensive studies of the so-called “pure” hydraulic fracturing gels based on viscoelastic surfactants began in the 1990s. These gels became known due to their complete destruction and subsequent cleaning of the hydraulic fracture.

Currently, there are a large number of reagent-thickeners (gelling agents) for aqueous and hydrocarbon media. As a rule, natural and synthetic polymers are used for aquatic environments, and there is a huge variety of such polymers. The former is most often subjected to various chemical modifications to increase the viscosity, thermal, and salt-resistant properties. There are significantly fewer thickeners for the formation of gels from hydrocarbon media. Most often, these are soaps of higher fatty acids and alkyl phosphates of aluminum and iron.

This section of the review will focus mainly on the chemistry and mechanism of the formation of hydraulic fracturing gels, as well as their destruction in various environments. We will also consider additional chemicals that are introduced into gels and their effect on the properties of compositions. We will identify the main features of various formulations based on the most common chemicals at the present time.

The review briefly discusses the main methods of studying the main process properties of hydraulic fracturing gels. The American Petroleum Institute (API) has developed a standard method for determining the properties of fracturing fluids API RP39 to assess the quality of various hydraulic fracturing gels. The methodology of the American Petroleum Institute includes methods for preparing hydraulic fracturing fluids in laboratory conditions and methods for studying their physico–chemical properties. In particular, it includes the determination of rheology, friction pressure losses, filtration, and sand retention capacity, for which special equipment is used. In addition, special techniques have been developed and used for the study of rheology, destruction of gels, filtration, determination of the restoration of rock permeability, and proppant packing, which are associated with specific testing equipment.

### 2.1. Guar-Based Gels

As mentioned above, there is a wide variety of polymer thickeners for the formation of gels. They have been used in hydraulic fracturing operations since the 1960s. Guar gum and its derivatives have become the most widespread gelling agents.

Using the example of this polymer in this section, we will consider the mechanism of gel formation and its destruction, as well as the action of various chemical additives in its composition. As described earlier, the destruction capability is an integral property of hydraulic-fracturing gels for the possibility of cleaning the fracture and the productive interval, as well as causing the inflow of formation fluids. However, the premature destruction of gels is also highly undesirable.

**Figure 2:**
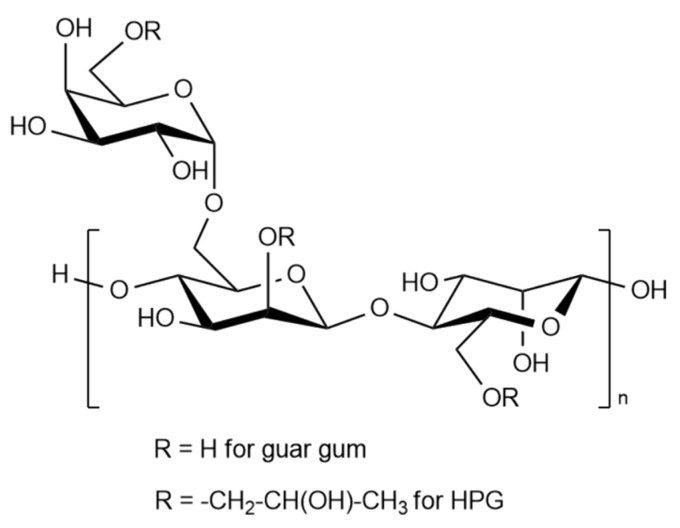
General structural formula for guar and its derivatives.

In addition to guar gum and its derivatives, other polysaccharides of plant and microbial origin have also become widespread:

1. Water-soluble cellulose derivatives (carboxymethylcellulose, carboxymethylhydroxyethylcellulose, hydroxyethylcellulose, etc.) [26,27]. Depending on the substituents, these polymers form gels in a wide range of viscosity, application temperatures, and mineralization of the water base. Heavy metal cations are often added to these systems to increase the viscosity.
2. Microbial polysaccharides: xanthan, emulsifier, simusan, kurdlan; bacterial alginates and fungal: aubazidan, pullulan, rodexman, scleroglucan. The most common of these is xanthan, which, due to its structure, forms highly viscous solutions in a wide range of pH, mineralization, and temperature. Guar and hydroxypropyl guar (HPG) are the cheapest of a biopolymer series, as well as effective thickeners of aqueous media for hydraulic fracturing. The general structural formula for guar and HPG is shown in Figure 2. The degree of polymerization of molecules (n) is usually 400–600, and the average molecular weight is in the range of 200,000 to 2,000,000 Daltons.

Hydration of polymer molecules occurs due to hydrogen interactions between polymer and water. Moreover, the introduction of substituents into the guar molecule with the formation of HPG increases the number of these interactions. The introduction of substituents into the molecule also increases the thermal oxidative and salt stability of the polymer. This is facilitated by the screening of polymer molecules by substituents introduced into the structure from oxygen dissolved in water, iron cations, and other metals, as well as from microorganisms.

When forming hydrogels for hydraulic fracturing, guar or its derivatives are dissolved in water to obtain low-viscosity solutions (viscosity about 100 mPa∙s) [26]. In this case, the so-called “linear gel” is obtained. Such a hydrogel can form a suspension of proppant only at sufficiently high concentrations. In addition, the linear gel is considered suitable for creating zones with high permeability near the borehole. It should also be taken into account that a low-viscosity linear gel, in addition to the difficulty with suspending the proppant, has another feature: large filtration leaks, especially in highly permeable collectors.

**Figure 3:**
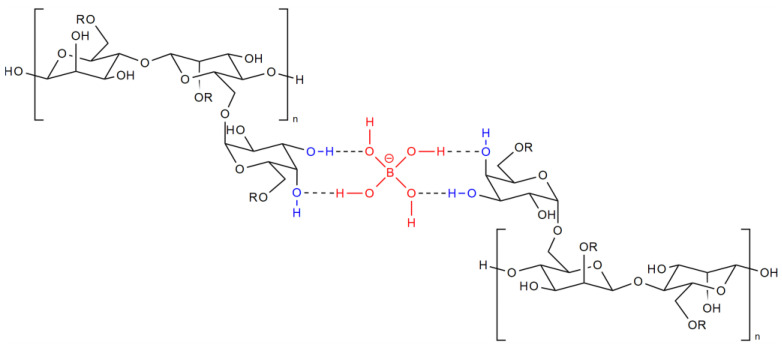
The structure of boron-linked guar. Blue shows the cis-hydroxyls of the polymer molecule; red shows the borate anion.

In the 1970s, complex elements such as boron, titanium, and zirconium were added to linear gels. Interacting with the polymer, they provide cross-linking of its links, and a “cross-linked polymer gel” is formed. Moreover, as shown in [28], cross-linking occurs at hydroxyl groups of guar, which are located in the cis position relative to each other (Figure 3). At the same time, the viscosity of the gel increases 5–10 times [29], which creates the ability to suspend and retain the proppant in the liquid volume, while filtration leaks into the formation also decrease.

The introduction of borate ions is possible in the form of water–alcohol solutions of boron compounds or suspensions [30,31], depending on the required cross-linking time. At the same time, it is necessary to create an alkaline medium for cross-linking (pH = 8.5), for which various buffer reagents can be used [32,33]. The use of other metals, such as titanium and zirconium [28,34,35,36,37,38], provides the resulting gels with a higher mechanical strength and thermal stability. Cross-linking takes place at a wider pH range. The quality of the cross-linking is strongly influenced by the composition of the water used, which should be pretreated [39].

**Figure 4:**
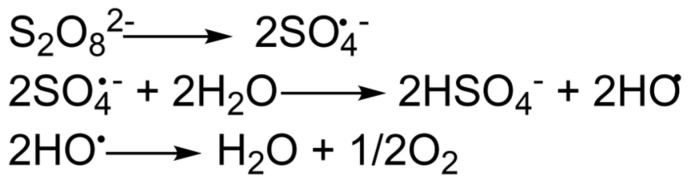
Active radical format ion from persulfate anion.

After the fracturing and propping the fracture, the fracturing gel should lose its viscosity. In other words, it should undergo destruction, as mentioned above. There is a wide variety of destructor reagents, depending on the type of systems. The destructor should perform its functions at reservoir temperatures, while the viscosity of the gel should not decrease much before the fracturing cracks are fixed. The most common destructors are oxidizing agents, such as persulfates or peroxides [40]. Moreover, the structure of these compounds justifies the possible temperature ranges of application. Destructors in the polymer shell are used to slow down the action at temperatures above 70–80 °C [41]. The action of oxidative destructors is based on the generation of free radicals that interact with the polymer chain and provoke its rupture to lower molecular weight components [42,43]. The generation of free radicals can be depicted by the example of a persulfate anion (Figure 4).

**Figure 5:**
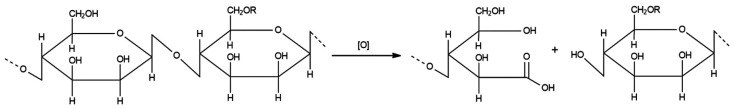
Scheme of oxidative destruction of polysaccharide.

The interaction of the obtained radicals with the polymer chain passes through hydrogen atoms, which are attached to the carbon skeleton of the polymer. Hydrogen atoms of hydroxyl groups in this case are less reactive. An example of destruction is shown in Figure 5.

Currently, enzyme destructors are often used, which undergo enzymatic cleavage of polysaccharide molecules [44]. Acids or reagents generating acids in situ conditions can be used as destructors in cases of borate cross-linking, which requires an alkaline medium. In this case, the polysaccharide–boron complex is destroyed [40].

Speaking of destructors, it is worth noting one of the features of guar-based gels. Currently, the issue of the completeness of the destruction of guar hydrogels is under discussion. The reduction of permeability by an undisturbed gel based on guar can reach 85%. Depending on the permeability of the collector, the quality of the colmatant can be affected by the types of salts present in the formation water and dissolved gases (hydrogen sulfide, for example) [45,46]. According to the researchers, a strong adsorption of polymer molecules also contributes to a decrease in permeability. It is proposed to reduce it by introducing various nanoparticles into the gel structure [47,48,49].

Guar gels, as well as hydrogels for hydraulic fracturing in principle, became widespread after the development of methods to reduce the negative impact of water systems on clays, which are an integral part of formations. Special reagent-stabilizers are used to prevent the swelling of clays (to stabilize them). Potassium and calcium chlorides have been used as clay stabilizers, while low-molecular-weight quaternary nitrogen-containing compounds, also called “ionic liquids”, are also used to prevent the swelling of clays [50,51].

It is worth noting that guar and its derivatives are subject to biodegradation. The mechanism of biodestruction is similar to the destruction under the action of enzyme destructors. Reagent-biocides are introduced to protect the system from the effects of microorganisms. Low-molecular-weight nitrogen-, sulfur-, or halogen-containing compounds are often used as biocide reagents, which completely suppress or inhibit the action of microorganisms [52].

In addition to these reagents, thermal stabilizers, friction reducers, and surfactants are additionally introduced into hydraulic-fracturing hydrogels in order to prevent the formation of stable oil–water emulsions, minimize capillary effects and changes in the wettability of the collector surface, as well as special corrosion inhibitors of steel, salt deposits, and asphaltene–resin–paraffin deposits.

Therefore, it can be concluded that guar-based hydrogel is a multicomponent system, and each component of the hydrogel performs certain functions to ensure the flow of fluids with minimal negative consequences for the formation rock and mining equipment.

### 2.2. Polyacrylamide-Based Gels

**Figure 6:**
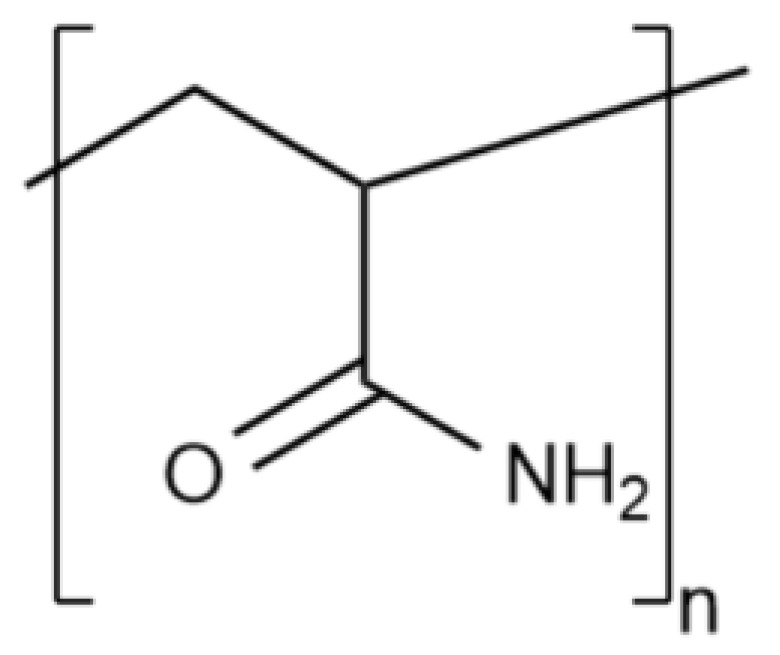
Structural formula of the PAM link.

Water gels based on a synthetic polymer—polyacrylamide (PAM) and its derivatives (also called slickwater) are widely distributed nowadays. The structural formula of the simplest PAM link is shown in Figure 6.

**Figure 7:**
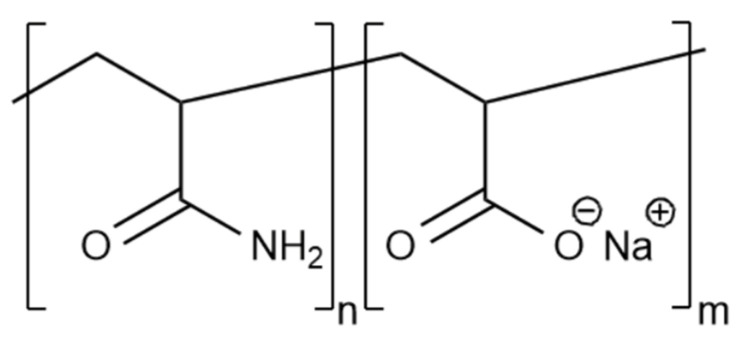
Structural formula of the copolymer of PAM and sodium polyacrylate.

In fact, PAM and sodium polyacrylate copolymers are most often used in hydraulic fracturing fluids (Figure 7).

The mechanism of hydration of PAM and guar is similar; they are hydrogen interactions between water and functional groups of the polymer. An increase in the degree of hydrolysis groups of the polymer molecule contributes to the production of solutions with higher viscosity values due to the repulsion of similarly charged functional groups, and, as a consequence, the opening of the polymer molecule.

One of the main differences between PAM gels and guar gels is the lower values of the “sand–bearing” viscosity: 100–200 mPa·s on average [6], at which the polymer solution is able to retain and transport the proppant. The method of oscillatory rheology is used for a more complete assessment of the properties of PAM solutions [6,7]. Based on the results of these studies, it is possible to most accurately assess the technological properties of these liquids: the ability to suspend proppant, resistance to thermal oxidative degradation, etc.

**Figure 8:**
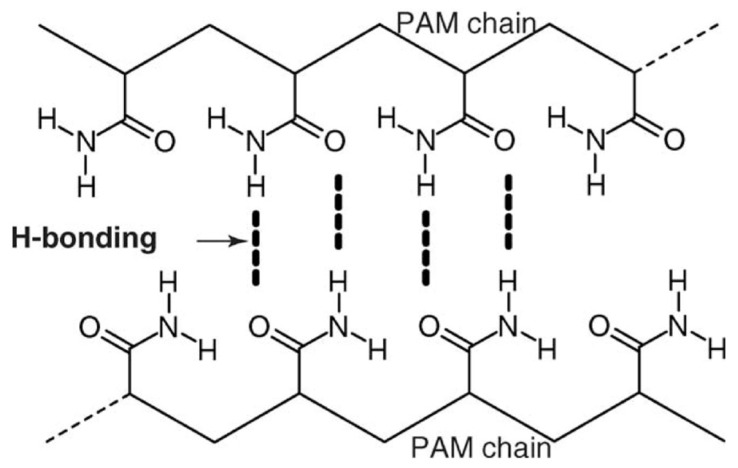
Hydrogen interactions between amide groups in PAM molecules. (Reprinted/adapted with permission from Deng et al., 2006, Ref. [9]).

Cross-linkers are usually not introduced in PAM-based gels. There are strong interactions between the functional groups of polymer molecules (Figure 8).

Due to this, the solutions have sufficient rheology for hydraulic fracturing without the introduction of cross-linkers. However, cross-linkers can be used to obtain thermally stable hydraulic-fracturing gels [8].

The PAM molecule contains functional groups that are reactive in themselves and affect the properties of the polymer chain. This is the reason for the peculiarities of these systems: sensitivity to thermal oxidative and salt degradation of PAM.

**Figure 9:**
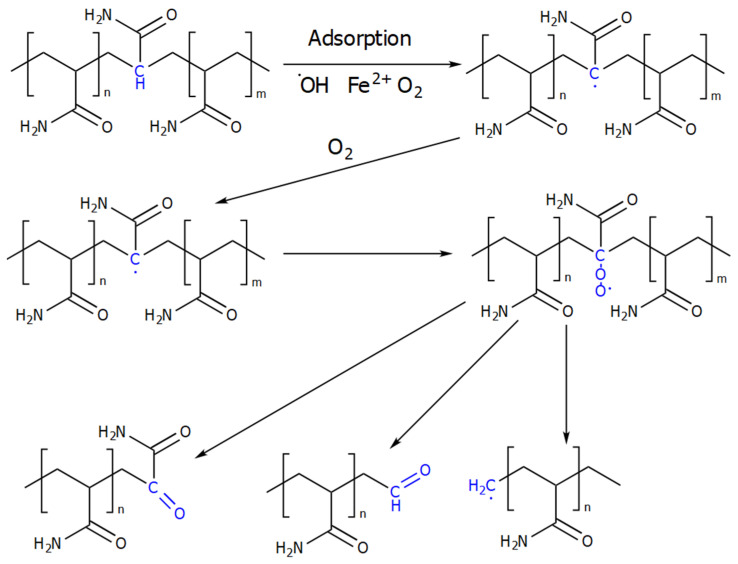
Scheme of thermal oxidative destruction of PAM. (Reprinted (adapted) with permission from Xiong et al., Ref. [10], Copyright 2018 American Chemical Society).

The scheme of thermo–oxidative destruction in the presence of iron salts is shown in Figure 9 [10].

**Figure 10:**
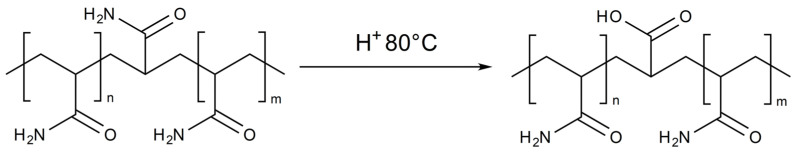
Hydrolysis of PAM.

High reservoir temperatures can also contribute to the degradation of solutions (Figure 10), especially in the presence of acidic media.

The rheology of solutions is also strongly affected by the presence of salts of monovalent and, to a greater extent, divalent metals [53].

Various substituents and copolymers are introduced into PAM molecules to improve the rheology of systems, as well as their thermal and salt resistance. Substituents can be hydrophobic radicals of various lengths, which are attached to the amide group. In addition, various substituents are introduced into the head groups in order to obtain cationic, anionic, and ampholytic polymers, which have a huge variety of properties. “Supramolecular” complexes consisting of surfactant–PAM associates are becoming widespread [54,55,56,57].

**Figure 11:**
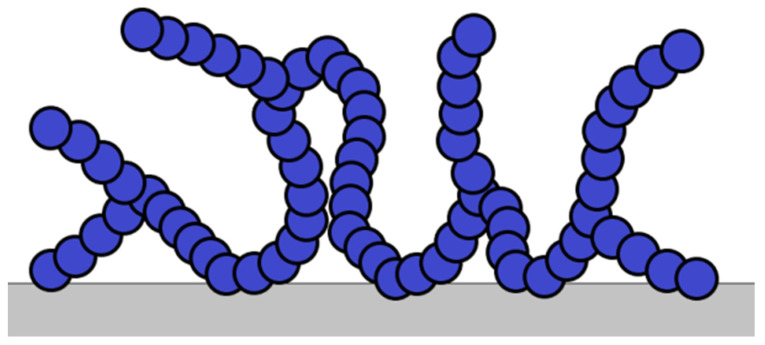
Polymer-adsorption scheme.

Functional groups of PAM are the reason for another feature: the tendency of the polymer molecule to strong adsorption (Figure 11), which goes through the stage of diffusion, fixation on the rock (metal equipment), and redistribution on it.

A positive consequence of this is that PAM minimizes friction pressure losses by adsorbing on the metal of the pipes. Adsorption on the rock leads to a decrease in the rheological characteristics of the solution, the filtration properties of the rock itself, and the destruction of the polymer [58]. PAMs are most susceptible to adsorption in acidic media [59]. However, studies [60] revealed that adsorption occurs mainly through hydrogen interactions with the rock. The authors suggest adding urea to hydraulic fracturing gels to reduce the adsorption of PAM.

**Figure 12:**
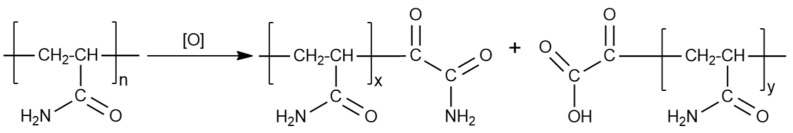
Scheme of oxidative destruction of PAM.

As in the case of guar gum, PAM-based liquids should be subjected to destruction after the hydraulic-fracturing operation. Mainly oxidative destructors are used for these purposes. Various persulfates, perborates, peroxides, hypochlorites, and their combinations have been used as destructors [61,62,63]. The destruction of these systems is effective, and colmatation occurs to a lesser extent than in the case of guar. The general scheme of PAM destruction is shown in Figure 12.

Clay stabilizers and various surfactants can be added to PAM gels in addition to these reagents, like in the case of guar gum. Usually, fewer reagents are used in these systems since most of the properties necessary for hydraulic fracturing can be achieved by chemical modification of the polymer itself.

### 2.3. Gels Based on Viscoelastic Surfactants

Fracturing fluids based on viscoelastic surfactants (VESs), or “pure” fracturing fluids, are new systems. Their intensive study has been conducted for two decades.

**Figure 13:**
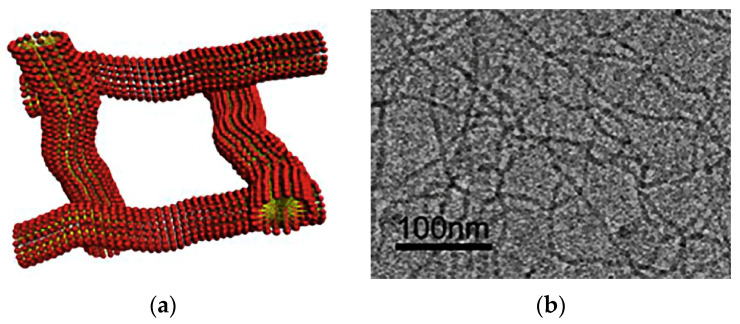
Micellar mesh microstructure: (**a**) Schematic image; (**b**) Photo. (Reprinted/adapted with permission from Yang et al., 2017, Ref. [64]).

A large number of VESs are known from the literature. They are used as the basis of viscoelastic compositions, while most VESs belong to the class of cationic surfactants (including dimeric [14,15,16,17] and trimeric [18] surfactants) and zwitterion surfactants [19,20,21]. They form associates in the aqueous medium in the form of long cylindrical micelles [17,22]. Solutions of cylindrical micelles are similar in properties of polymer solutions. Long cylindrical micelles are capable of forming a three-dimensional interlaced grid in a solution (Figure 13), due to which the solution acquires viscoelastic properties.

Unlike polymer chains, worm-like micelles of VESs are dynamic structures. They are also called “living polymers.” The micellar chains of the VES are reversibly destructed under mechanical impact and then restored. Significant changes in the structure of the micelles of the VES are observed with a slight change in the thermodynamic parameters, which affects the rheology of the solution, phase behavior, etc.

We will highlight some features of hydraulic-fracturing gels based on VES:

- Relatively low viscosity values, which contribute to the formation of a long conductive fracture [17,65,66].
- High elastic properties in fresh and mineralized water, due to which the fracturing fluid has the necessary sand-carrying capacity [15,21].
- Oil-flushing properties, which can increase the oil recovery factor (ORF).
- Hydrophobization of the formation rock due to the adsorption of surfactants. This contributes to the stabilization of clays, and also prevents the formation of water blockades after hydraulic fracturing [67].
- Destruction of the structure in contact with reservoir fluids, which contributes to the complete restoration of the permeability of the rock after treatment [15,21,68].

As in the case of PAM-based compositions, oscillatory rheology is an important criterion for evaluating fracture fluids based on surfactants.

VES-based compositions are characterized by high values of elastic properties (modulus of elasticity/accumulation). The viscoelasticity of the compositions can provide fracturing fluids with an optimal mechanical strength, as well as a good ability to retain the proppant in volume. Well-known studies show that the “sand-bearing” viscosity of surfactant-based compositions is lower than compositions based on polymer gels [69,70]. The energy is more efficiently transferred from the well head to the bottom due to the elastic component when using surfactant systems as fracturing fluids, which can reduce the energy consumption during hydraulic fracturing.

**Figure 14:**
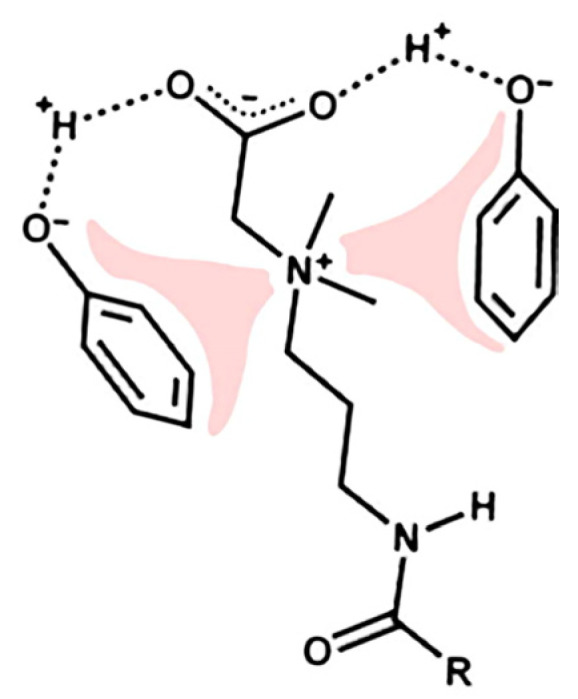
Schematic representation of intermolecular interactions between surfactants and polar hydrocarbons on the example of a zwitterion surfactant and phenol. (Reprinted/adapted with permission from McCoy et al., 2019, Ref. [72]).

Usually, the use of VES-based fluids eliminates the need to introduce a destructor. Worm-like micelles in contact with petroleum hydrocarbons solubilize them. At the same time, the volume of micelles increases until such a state ceases to be energetically advantageous. Then, the micelles break up into smaller aggregates. As a result, the viscosity of the composition decreases sharply [71]. This process runs in parallel with another: a long hydrocarbon radical is most often present in the VES molecule, which has an affinity for petroleum hydrocarbons. A certain number of surfactants can pass into the oil phase as a result. The viscosity of the aqueous medium of the VES will simultaneously decrease. However, it is necessary to investigate in each specific case how this will affect the properties of oil. It was shown in [72] that VESs are capable of entering into very strong interactions with hydrocarbons containing polar functional groups (Figure 14). Such components are present in almost any oil.

In rare cases, researchers propose destructors for surfactant-based compositions. For example, in the work considered earlier [71], for the destruction of the “hook-like” dimeric VES, the authors propose using a strong oxidizer, such as ammonium persulfate, which breaks the surfactant molecule at double bonds. The length of the hydrocarbon radical decreases and, as a result, hydrophobic interactions decrease.

Destruction in case of contact with reservoir fluids and the absence of polymer in the system ensures almost a complete recovery of the conductivity of the proppant batch and the fractures formed after the hydraulic-fracturing operation. Therefore, fracturing fluids based on surfactants are called clean fracturing fluids [69,70,73]. However, the absence of a polymer in the system justifies a significant drawback of this type of liquid: filtration leaks in reservoirs with a permeability of more than 100 mD. Large filtration leaks of VES-based systems are attributable to the fact that such liquids do not form a sufficiently pronounced filter cake that prevents filtration leaks. A polymer is added to the fracturing liquid based on surfactants to avoid filtration losses, as, for example, in [74]. The authors conducted a study of a mixed system based on cationic surfactant and xanthan. It was found that a filter cake is formed during filtration through a porous medium, which prevents filtration leaks. The filter cake is removed with the subsequent injection of oil, and the permeability is almost completely recovered.

Surfactant compositions are rarely used in an individual form. Structure-forming reagents are introduced into almost any system, which contribute to the improvement of the structural and mechanical properties of the compositions. Surfactants containing two or three hydrophobic tails at once are increasingly being considered as new, modified high-tech systems [75,76]. Various electrolytes [77], surfactants of another class [78], polymers [79], and nanoparticles [80,81] can act as structure-forming reagents.

### 2.4. Hydrocarbon Gels

Hydrocarbon gels were used at the first stages of the development of the hydraulic-fracturing process, but they are also currently used in high-temperature and water-sensitive formations. Soaps of higher fatty acids are used among the hydrocarbon liquids for hydraulic fracturing at low temperatures, and aluminum or iron alkyl phosphates are used at high temperatures. However, carboxylic acids are known as destructors at low temperatures, as well as medium (NaHCO_3_ and CaO) and high organic amines. The best filtration reducers are oil-soluble polymers, benzoic acid, ground naphthalene, or inorganic salts [11].

**Figure 15:**
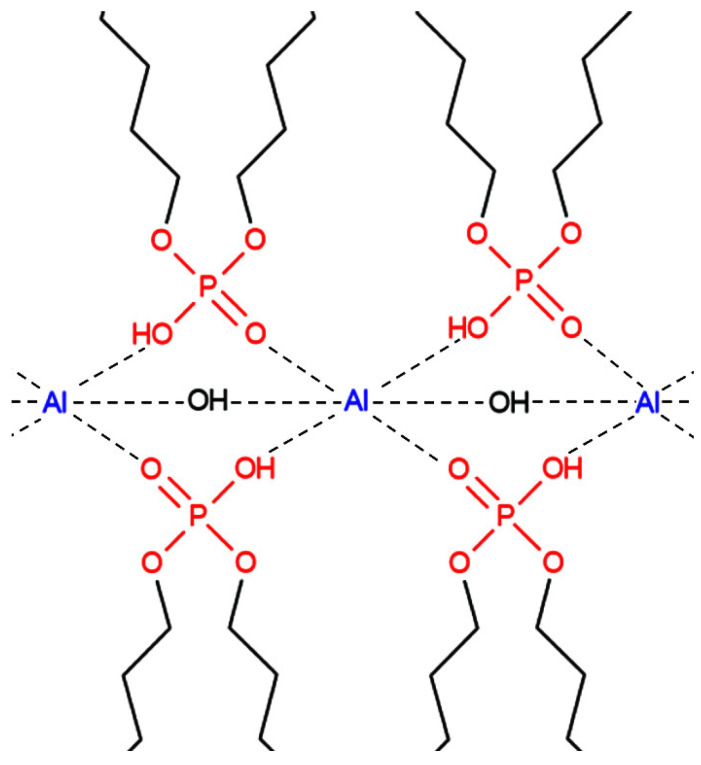
Structural formula of aluminum alkyl orthophosphate associates.

Aluminum and iron alkyl orthophosphate soaps are the most promising hydrocarbon gels for hydraulic fracturing in terms of the stability of structural, mechanical, and rheological properties at high temperatures. Associated complexes of significant molecular weight are formed, coordinated by intermolecular hydrogen bonds produced when these forms of thickeners are dissolved in hydrocarbons (Figure 15) [12,13].

**Figure 16:**
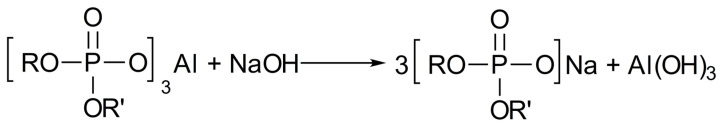
Destruction mechanism of orthophosphorus ester complexes.

Dry inorganic compounds of an alkaline nature (for example, sodium carbonates and bicarbonates) are used as a destructor of these gels. They are hydrolyzed in case of contact with water contained in the reservoir fluid producing an alkali, which, interacting with aluminum salts of organic orthophosphoric esters, forms sodium salts and destroys the gel complex (Figure 16).

The exchange reaction also occurs in an anhydrous medium, but the presence of water accelerates the rate of destruction. Therefore, increased requirements are imposed on the water content in the hydrocarbon liquid used for gelling; its amount should not exceed 1%. In case of filtration during hydraulic fracturing, the particles of the destructor mainly remain in the fracture. The gel, filtered into the formation due to the lack of the destructor, clogs the pores for a long time.

To conclude this section, let us note the main features of each type of fracturing gels. Gels based on guar gum and other polysaccharides are currently the most common. This is due to their low cost, ease of preparation, and eco-friendliness. However, despite the various modifications of polysaccharides, this type of gel still has a complex composition. Incomplete destruction of the gel system in reservoir conditions makes research on the development of destructors that will provide a complete restoration of the porous media permeability relevant.

Gels based on synthetic polymers such as polyacrylamide and its derivatives solve the problem of the multicomponent nature of the previous type of fluid. PAM is much more resistant to various environmental conditions, and its properties can be easily varied through chemical modifications of the polymer chain. For these gels, the issue of possible filtration leakage of fluid is relevant; various fillers are proposed to solve this problem. The chemical resistance of PAMs leads to the need for stronger destructors than for polysaccharide-based gels.

Surfactant-based compositions are the most modern direction of research in hydraulic-fracturing technologies. The variety of surfactants allows for the selection of systems practically for almost any medium and condition. Surfactants in these compositions perform several functions at once: gelling agent, clay stabilizer, demulsifier, rock hydrophobizing agent, lowering interfacial tension at the boundary with hydrocarbons, etc. The relevant direction of research for these systems is the selection of various modifier reagents, the use of which is aimed at reducing the working concentration of surfactants in gels to reduce composition costs while maintaining necessary rheological properties.

Hydrocarbon gels were the very first fracturing systems. However, their use is becoming increasingly rare due to increased safety requirements for their use, as well as high environmental risks. Of the listed systems, hydrocarbon gels have the least damaging effect on the reservoir and crude oil, which is why their use remains relevant.

## 3. Gels for Conformance Control and Flow Diversion

Gels for conformance control and flow diversion are almost identical in composition and are based on the same reagents. The term “flow diversion” is usually used to emphasize the significance of the volume of injection of the gel-forming composition and regulation of water filtration over the area of the site, and the term “conformance control” is used to denote the regulation of water filtration along the section and refers to injectivity profile data before and after gel injection. We will use both terms depending on the formulation of the problem being addressed.

### 3.1. Gels Based on Acrylamide Polymers

**Figure 17:**
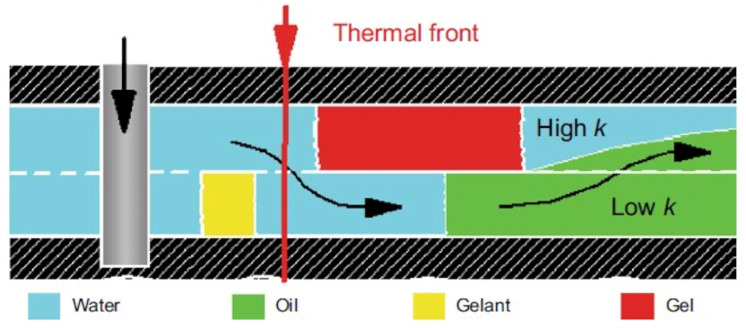
Illustration of in-depth profile modification [1].

Historically, gels based on partially hydrolyzed polyacrylamide, as well as chromium and aluminum salts, were used as one of the first compositions for conformance control, and an interest in these gels has not decreased at the present time [1,82]. Due to the adjustable cross-linking time and the transition of the polymer solution into the gel, it is possible to place the gel screen at a predetermined distance from the injection well in such a way that, by changing the direction of the water flow, it leads to the displacement of oil from the bypassed parts of the reservoir (Figure 17).

**Figure 18:**
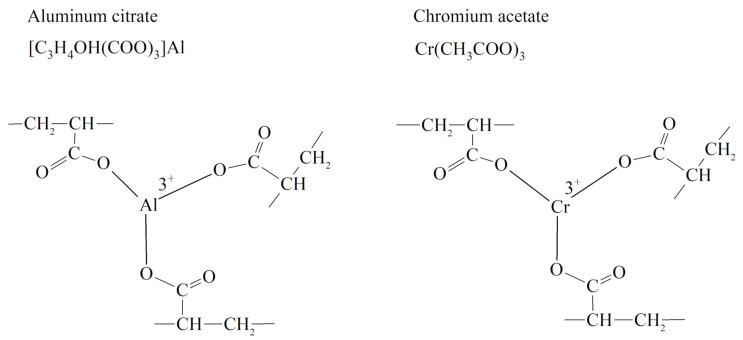
Cross-linking of partially hydrolyzed polyacrylamide with ions of trivalent metals [83].

Trivalent metal salts act as cross-linkers in this technology: when they interact with the carboxyl group of the polymer, a cross-linked spatial structure is formed that prevents water filtration [83] (Figure 18).

Such hydrogels are called cross-linked polymer compositions (CLPC). The gelling time, as well as the structural and mechanical properties of the CLPC, is selected depending on the reservoir temperature by varying concentrations of components.

The CLPC treatment of layers with high layer-by-layer heterogeneity in permeability (≈10:1) and large values of the thickness ratio of the layers of different permeability (low-permeable intervals are ≈10 times thicker than high-permeable ones) is especially efficient. Deep treatment of the bottom-hole zone of the formation allows for the redirection of the water flow from a highly permeable interval to a low-permeable one (Figure 16). Due to the different filtration rates of the unformed gel (gelant) in the intervals of different permeability after gelling, the water flow bends around the barrier in the highly permeable part of the reservoir and displaces oil from the low-permeable part.

**Figure 19:**
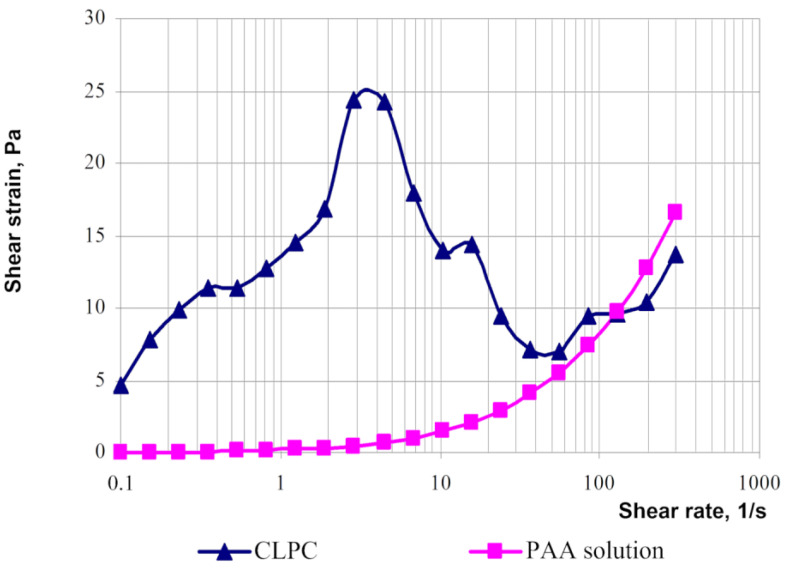
Typical flow curves of polyacrylamide solution and cross-linked polymer composition based on PAM [23].

The rheological behaviors of solutions of linear polymers and CLPC-forming gels differ significantly [23]. For instance, the rheological curves of the gels have an extreme character if the shear strain for polymer solutions increases parabolically with an increasing shear rate (Figure 19).

**Figure 20:**
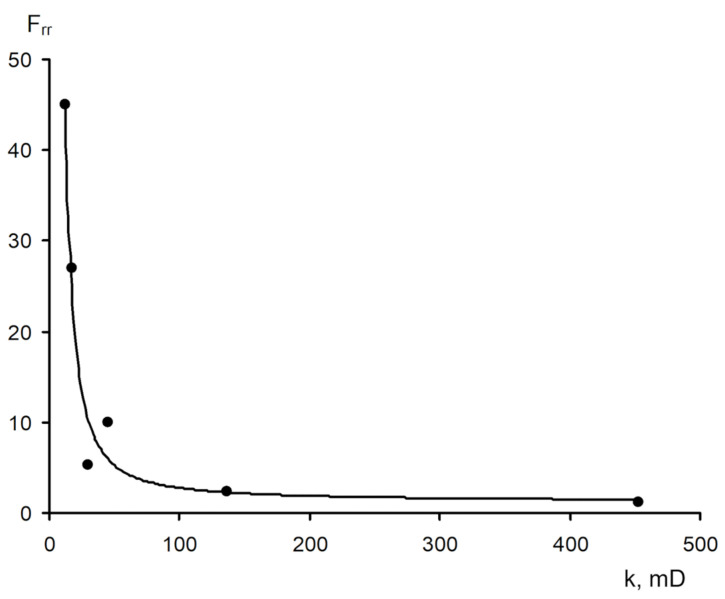
Relationship of the residual resistance factor (Frr) and permeability (polymer concentration with 0.06 wt.%) [23].

**Figure 21:**
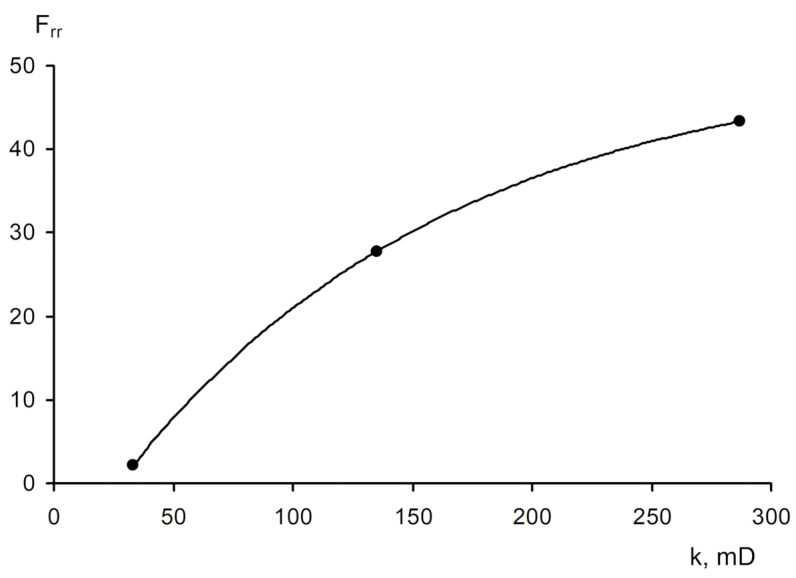
Relationship of the residual resistance factor (Frr) and permeability in case of filtration of CLPC (the size of the slug is 0.3 pore. vol.) [23].

The area of shear strain growth to the maximum value corresponds to an unbroken cross-linked structure. The spatial structure is destroyed with a further increase of the shear rate. The strain at the inflection point represents the “ultimate strength”, and the corresponding shear rate represents the “critical deformation”. Such cardinal differences in rheological behavior in polymer solutions and gel structures also lead to a fundamental difference of their filtration characteristics [23]. If the residual resistance factor, calculated as the ratio of the pressure drop in water after and before the injection of polymer and gel slug, decreases hyperbolically with increasing permeability in case of linear polymer solutions, then by contrast, it increases in the case of cross-linked gel structures [23,84,85] (Figure 20 and Figure 21).

**Figure 22:**
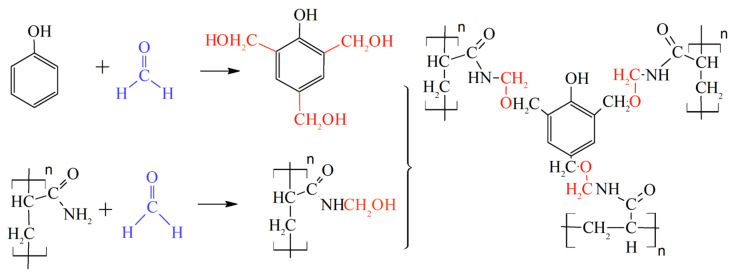
Covalent bonds formed by HPAM and phenol/formaldehyde. The cross-linking mechanism includes two steps: (1) Hydroxymethylation of the nitrogen on the amide functional group; and (2) Cross-linking with multiple alkylations on the phenol ring. (Reprinted (adapted) with permission from Zhu et al., Ref. [88], Copyright 2017 American Chemical Society).

A lot of theoretical and laboratory studies address the properties of CLPC of various nature and composition [86,87,88]. However, polyacrylamide-based hydrogels with a Cr^3+^ or Al^3+^ cross-linking are still the most popular in commercial practice. It should be noted that the use of organic staplers—a mixture of phenol and formaldehyde—has allowed for the expansion of the boundaries of the applicability of gels in high-temperature layers, since the cross-linker protects the polymer from thermal oxidative destruction (Figure 22, [88]).

**Figure 23:**
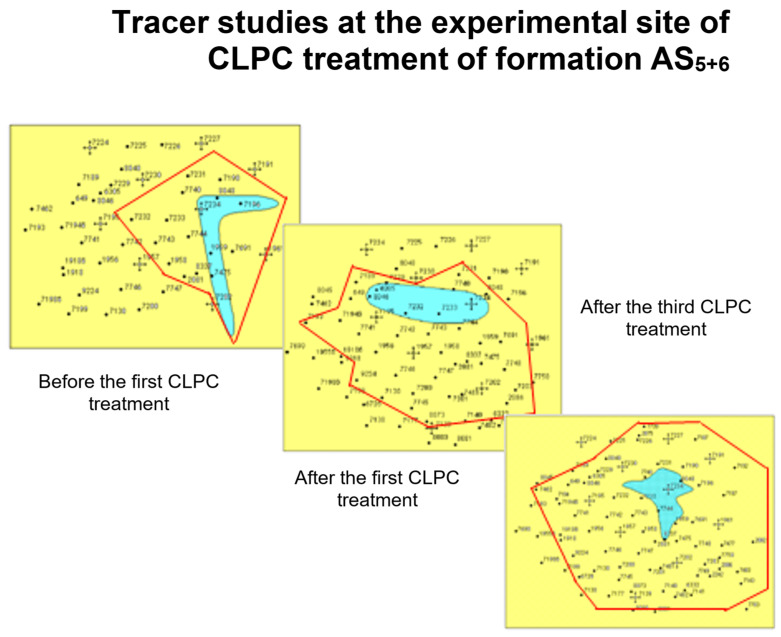
Indicator studies in the experimental area of application of the CLPC in formation AS_5+6_.

In commercial practice, the injection of sufficiently large volumes of polyacrylamide with a cross-linker (≈10 thousand m^3^) in concentrations corresponding to the lower boundary of gel formation allows for the deep treatment of the bottom-hole zone of the formation and significant redistribution of filtration flows [84,89]. Therefore, Figure 23 shows that there was actually a linear flow of water from the injection well in two perpendicular directions before CLPC treatment according to the tracer study data. There was a noticeable redistribution of the flow after the first injection of CLPC, and the flow became radial after the third treatment [23].

Mixtures of water-soluble cellulose esters and polyacrylamide are also capable of forming gels under reservoir conditions under the impact of a cross layer with an adjustable gelling time [90]. Gels based on hydrolyzed polyacrylonitrile with formalin and hydrochloric acid are used in carbonate fractured reservoirs. Moreover, in this technology, formalin is the cross layer of the polymer, and hydrochloric acid is the initiator of the gelling reaction [90]. Hydrolyzed polyacrylonitrile in carbonate-fractured reservoirs is pumped without any additives. The polymer is cross-linked with the formation of gel deposits by reaction with ions of alkaline earth metals contained in reservoir water [91]. In addition, already-formed polyacrylamide-based gels and a cross-linker are injected into carbonate reservoirs to isolate fractures from injection wells, which are not filtered into the pore matrix but selectively shut off only the fracture conductivity of the formation [1].

Gel systems containing water-swellable yet insoluble particles of cross-linked polymers (gel-particle dispersions) are of great interest for flow-diversion purposes. The synthesis of water-swellable polymers can be carried out in various ways:

− At the polymerization stage, the bifunctional monomer methylene bisacrylamide is introduced into the composition of acrylamide and acrylic acid monomers [92].
− Heat treatment of polyacrylamide at moderate temperatures when the cross-linking of macromolecules occurs as a result of the imidization reaction [92].
− Radiation cross-linking of powdered polyacrylamides by gamma or beta radiation [93,94,95,96,97].

The gel fraction is a particle with a three-dimensional cross-linking; it is capable of swelling up to 1000 times. Such systems containing dispersion of gel particles are able to significantly reduce the permeability of water-conducting, highly permeable porous and fractured interlayers, and the presence of a linear polymer bearing the gel fraction ensures a better filterability, provides viscoelastic properties, and increases the penetration depth of the flow diversion composition into the formation [98,99,100].

**Figure 24:**
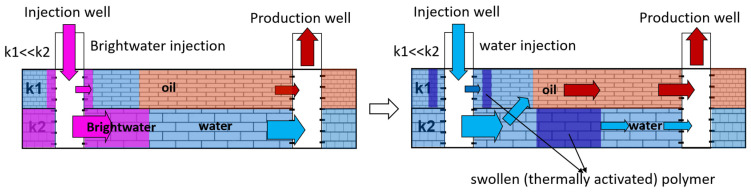
Schematic diagram of oil displacement using Bright Water injection.

The injection of microparticles of cross-linked polyacrylamide obtained by emulsion polymerization after thermal activation in the formation allows for the formation of flow-diversion gel screens at a particular distance from wells. Polymer microparticles of ≈0.5 microns in size are injected as a dispersion in an organic solvent and forced through the formation. Due to the reservoir temperature in the water, the particles swell, after which they lose the ability to filter. This technology was named BRIGHT WATER and was developed by a consortium of SHEVRON TEXACO, BP, and Nalco Company in 1977 [101,102]. A schematic diagram of oil displacement in an inhomogeneous reservoir by intra-reservoir flows is shown in Figure 24.

### 3.2. Gel-Dispersed and Sedimentary-Gel-Forming Compositions

**Figure 25:**
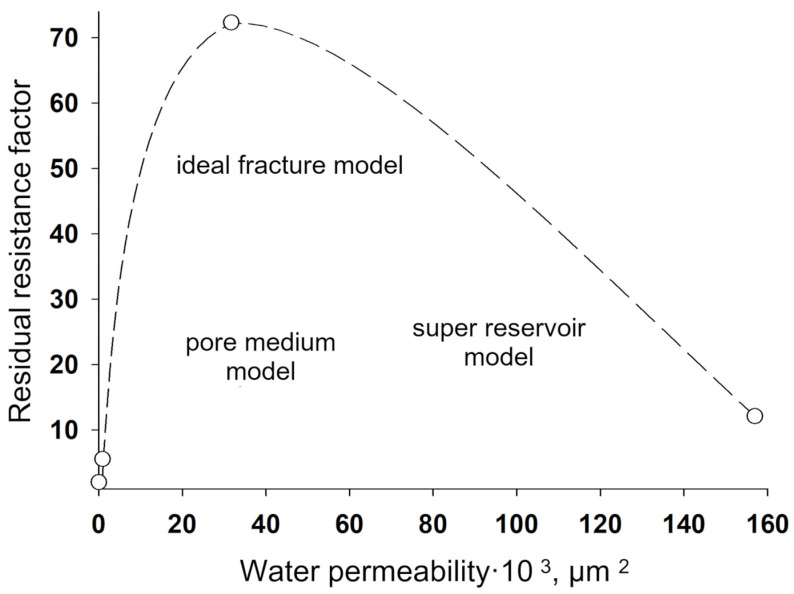
Dependence of the residual resistance factor for modified polyacrylamide cross-linked with aluminum oxychloride on the initial (before treatment) permeability of the medium to water.

The transformation into sedimentary-gel-forming and gel-dispersed materials by introducing dispersions (clay, chalk, marl, and wood flour) has been one of the remarkable trends in the development of conformance control technologies using gels in Russia over the past 10 years, which enhances the structural and mechanical properties of the system, allowing for the reduction of the volume of its injection [23,103,104]. Sedimentation and gel-forming systems are obtained by the interaction of aluminum oxychloride with modified polyacrylamide [105]. Such gel additives also have selectivity in permeability, i.e., with increasing permeability, the residual resistance factor also increases (Figure 25).

Figure 24 shows the values of the residual resistance factor during filtration of the sedimentary-gel-forming reagent in three media differing in permeability and structure of the pore space: pore medium, super reservoir, and fracture. It can be seen that in the pore medium, the residual resistance factor is minimal compared to the super reservoir and the fracture model.

**Figure 26:**
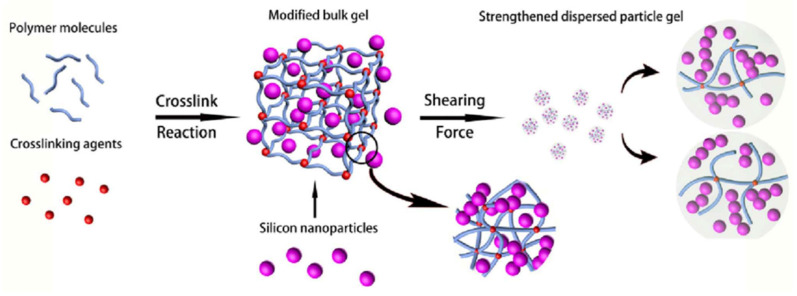
Schematic of strengthening gel particles by nano-silica particles. (Reprinted/adapted with permission from Dai et al., 2016, Ref. [106]).

The original solution to increase the thermal stability of the CLPC is described in [106] and the review [88] (Figure 26).

However, not only the thermal stability of the gel increases, but also the resistance to salt aggression of formation water.

### 3.3. Gels Based on Inorganic Compounds

Methods of regulating intra-layer filtration flows using inorganic gels allow for the creation of strong barriers to water filtration, which leads to a change in the direction of movement of the displacing agent and to the connection of oil-saturated, poorly drained, untreated interlayers to development [3]. Gels based on silicon compounds, such as silicates and aluminosilicates, have become the most widespread. Silicate gels are formed by the acidification of alkaline solutions of sodium silicate with acids to neutral pH values. The silicate gel, being a pseudoplastic material, “breaks” with the formation of microgel agglomerates ranging in size from 2 to 27 microns when injected into the injection well and advancing through the formation. Such a microgel dispersion in sodium silicate solutions has both viscoplastic and viscoelastic properties, which allows for the redirection of water flows from the developed high-permeable intervals to low-permeable, oil-saturated ones [107,108].

Polysilicic acid gels, for leveling the conformance control, are also obtained from aluminosilicate, a natural mineral of nepheline [109]. The principle of the technology is that the aluminosilicate, when dissolved in inorganic acids, forms a composition that is able to coagulate, turning into a gel. Dissolution occurs with an excess of acid. Subsequent gelling occurs by aggregation with the formation of three-dimensional polymer meshes. The initial particles condense together with an increase in the concentration of the solution, forming a “ringing” gel. As a result of the interaction of aluminosilicate with hydrochloric acid, an aluminosilicate sol is formed, followed by a monosilicic acid, its low-molecular-weight oligomers, and then its silicic acid sol, which turns into a gel [110].

Solutions of other acids, such as phosphoric and sulfamic acids, can also be used in gel-forming compositions [111,112,113]. In addition, synthetic zeolites are used as aluminosilicates [114].

The use of bicalcium silicate allows for the accumulation of acidic gels with an adjustable gelling time, which makes it possible to place the flow diversion material at a particular distance from the well [115,116].

It is proposed to add partially hydrolyzed polyacrylamide to the composition to provide gels based on sodium silicate with viscoelastic properties [117]. A similar technique for obtaining a silicate–polymer gel is described in [118]. Only the spent catalyst Zeokar-10 was used as a source of aluminosilicate after dissolving it in a weak alkali. Water-soluble cellulose derivatives were also used as a polymer, along with polyacrylamide.

L. K. Altunina and her co-researchers experimentally substantiated and implemented the technological process of using both inorganic and organic gels for well conformance control for deposits characterized by high layer-by-layer heterogeneity and temperature. These are gel-forming systems that are low-viscosity solutions in surface conditions and turn into gels in reservoir conditions. The factor that causes gelling is the thermal energy of the reservoir or the injected coolant. Gel-forming compositions with different gelling times—from several minutes to several days—in the temperature range of 40–120 °C were proposed. These include inorganic gel-forming compositions GALKA, GALKA–PAV, and GALKA–U based on the system “aluminum salt—urea—water,” as well as compositions METKA based on thermally reversible polymers of methylcellulose [4,119,120,121].

GALKA and GALKA–PAV surfactants are low-viscosity solutions with pH = 2.5–3. They contain aluminum salt, carbamide, and some additives that improve their process parameters. The urea is hydrolyzed, forming ammonia and CO_2_ due to the thermal energy of the reservoir or the energy of the injected coolant, which gradually increases the pH of the solution. The aluminum hydroxide gel is formed in the entire volume of the solution at pH = 10. This is manifested in an abrupt increase in the dynamic shear strain of the gel-forming solution.

The gels formed by the METKA compositions are reversible. When the temperature decreases, they can turn back into a liquid. When the temperature rises, they can form a gel again, which makes it possible to “open” and “close” the interlayers by changing the temperature to regulate filtration flows. This property of gels can be used for a cyclic injection of hot water or steam in order to increase the coverage of the reservoir by thermal action. Currently, thermotropic inorganic gel GALKA is used with steam-assisted gravity drainage (SAGD) technology for steam diversion in horizontal wells [122].

Thus, various gel-forming compositions based on water-soluble and water-swelling polymers, as well as inorganic silicon and aluminum compounds, are used for flow diversion and conformance control. The combination of gels with dispersants and precipitating compositions allows for the enhancement of the structural–mechanical properties of the diversion system, making it possible to apply these compositions in naturally fractured reservoirs. The ability to regulate the gel formation time allows for the placement of the gel screen at a specified distance from the injection wellbore. This approach has firmly entered industrial practice and continues to be improved both in terms of reducing the cost of the compositions used and increasing their effectiveness.

## 4. Gels for Water and Gas Shutoff

### 4.1. Gels Based on Organic Polymers and Inorganic Compounds

Remedial cementing is of particular importance in the conditions of intensive watered wells and aged main well stock, while most of the remedial cementing operations today cannot be imagined without the use of gels [24,123,124].

It is known that the wells’ watering is caused by such factors as the rise of oil–water contact, the inflow of injected and edge water through highly permeable interlayers or fractures, production casing leaks, water coning, and behind-the-casing cross-flows [125,126]. Water shutoff technologies based on gels capable of forming blocking screens in flooded areas of the formation are used depending on the task being addressed, the purpose of which is to shut off highly permeable water-saturated layers from active development. If necessary, the gel screen is reinforced with a more rigid grouting compound based on curing resins or microcement, which are pumped after the gel-forming composition.

The treatment of watered interlayers with cross-linked polymer compositions based on polyacrylamide and chromium salts, which we have already mentioned when describing the injection of such compositions for conformance control, has long become a classic technology. The main difference between water shutoff compositions is a higher concentration of reagents that allow for the formation of “strong” gels (according to the terminology of R. Seright, the world’s leading expert in this field) so that the flow of fluids into the well does not tear or pierce through the water shutoff screen.

The work [124] describes the study of the development of selective water shutoff gel-forming compounds based on polyacrylamides and polyatomic phenol alcohols. The authors of the article [127] used a mixture of paraform and resorcinol as a cross-linker to increase the strength and thermal stability of polyacrylamide gel. This hydrogel has passed comprehensive laboratory testing and commercial tests, which allowed it to gain steady positions in the field operations [128,129].

Another example of a gel formed by cross-linked covalent bonds is shown in [130], which is obtained by the interaction of polyethylene glycol and polyvinylpyrrolidone. N,N′-methylene-bis-acrylamide is used as a cross-linker, and ammonium peroxodisulfate is used as the initiator of gelling. Polyvinylpyrrolidone is used in the system to increase the strength of the gel. Gelling occurs at temperatures from 25 to 100 °C in a time interval from 6 to 60 h.

The results of studies of gels based on polyvinyl alcohol and polyvinylpyrrolidone, in which a mixture based on resorcinol and formaldehyde was used as a cross-linking agent, are of interest. Viscoelastic and thermal properties of the mixtures were studied by oscillatory rheometry and differential scanning calorimetry. The results of rheological studies have shown that the developed gel systems are viscoelastic since the values of the elastic modulus of the studied samples are higher than the values of the loss modulus (G′ > G″). The content of free and bound water in the gels was determined, as well as their thermal stability at temperatures up to 90 °C based on the results of calorimetric studies. The effectiveness of the prepared gel-forming compositions for isolating the water inflow was tested on bulk reservoir models (super reservoir model). During the experiment, a noticeable decrease in permeability was shown in the case of the use of all the proposed gel-forming compositions [131].

Compositions based on liquid glass have been used for a long time as a water shutoff gel [132,133,134]. Such gels are characterized by good filterability in the pore space, controlled setting time, high values of the limiting shear strain, and the ability to form homogeneous mixtures with various reinforcing additives.

**Figure 27:**
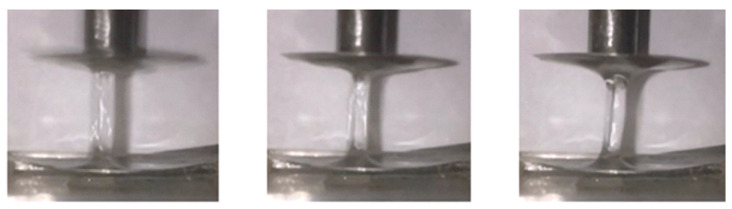
Formation of liquid hydrogel filament (filament lifetime ~0.45 s).

An interesting development of gel technologies using liquid glass was obtained in the works of V. N. Duryagin [135], in which the initiator of gelling—mineral acid—was replaced by Lewis acid. Polycondensation of silicic acids under the action of chromium acetate leads to the production of strong ringing gels with an adjustable gelling time, which makes it convenient to use them in field operations. Acidic silicate gels based on natural nepheline material, as well as synthetic zeolites or waste from production, allow for relatively cheap waterproofing materials to be obtained [136,137,138,139]. A distinctive feature of the rheological behavior of silicate gels is that they have viscoplastic properties with high values of the ultimate shear strain. However, they also lack viscoelastic properties necessary for a high-quality water shutoff. In [140], partially hydrolyzed polyacrylamide in an amount of only 0.05% was introduced into the sodium silicate–chromium acetate system to impart viscoelastic properties to the silicate gel. The authors of this article used a relaxometer for the primary analysis of the viscoelastic properties of the obtained hydrogels. The mechanical part of this device consists of two disks in the space between which the gel understudy is placed. The upper disk is quickly raised by a spring mechanism during the study, colliding with the locking mechanism. Under the action of the spring, it returns to its original stationary position. The lifetime of the thread, which to some extent is a measure of the viscoelastic properties of the gel, was determined automatically by recording the time during which there is electrical conductivity between the upper and lower disks of the relaxometer. An illustration of the process of forming a liquid filament of a three-component hydrogel is shown in Figure 27.

It was determined that a liquid bridge is not formed for a highly viscous gel-forming composition consisting of liquid glass and chromium acetate without polymer.

Rice-husk dispersion was introduced into the composition of the three-component hydrogel to increase viscoelastic properties [141]. Previously, this dispersion was used for water shutoff in silicate compositions [142,143]. It was determined that mechanically activated rice husk increases the stability of hydrogel during filtration in cracks. According to the results of rheometry, it was found that the addition of a rice husk to the gel in question increases the safety of its undisturbed structure with an increase in shear strain while also increasing the yield strength.

Rheological measurements (flow curves, viscosity curves) of this gel were carried out before and after filtration through a model of an ideal crack with a different opening of 0.01 to 0.1 cm. It was determined that the intensive destruction of the insulating material occurs in fractures with an opening of 0.01 cm. The addition of rice-husk dispersion leads to a significant increase in the resistance of the hydrogel to mechanochemical destruction. The addition of 0.1% rice husk reduces the difference in effective viscosity by an order of magnitude before and after filtration through a 0.01-cm opening. There was no noticeable difference in rheology of a hydrogel in the case of its filtration through a 0.1-cm opening with the rice husk. Without it, only a slight strengthening of the gel is noticeable after filtration at low shear rates. This behavior of gels is explained by the fact that the geometric dimensions of the crack reformat the structure of the insulating material during filtration [144]. Supramolecular formations provide the necessary complex of properties of hydrogels. They also deform and collapse in small fractures, and they are preserved in large fractures. In turn, dispersed rice-husk particles strengthen the hydrogel, contributing to the preservation of the structural and mechanical properties of the hydrogel [145].

The results of experimental work indicate that large fractures that account for the greatest inflow of water will be most reliably isolated. Apparently, the addition of rice husks strengthens the hydrogel structure due to the flocculation of dispersed particles by polymer macromolecules.

Quantitative representations of viscoelastic properties are provided by oscillatory measurements. In particular, this includes creep and recovery tests and their interpretation using the Maxwell, Kelvin–Feucht model, as well as the Burgers model made up of them. A single Kelvin–Feucht link is not enough to approximate the data of real measurements. Here, a two-component Burgers model is used, which corresponds to two relaxation times.

**Figure 28:**
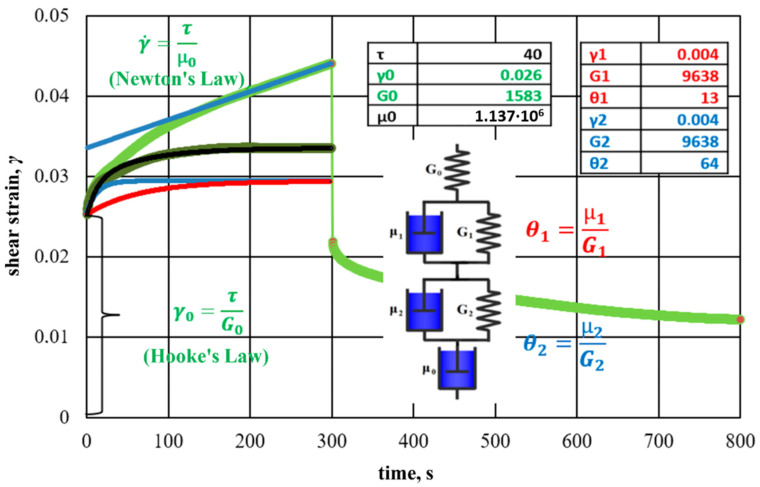
The results of the creep-and-recovery test of a hydrogel based on sodium silicate, polyacrylamide, and chromium acetate with the addition of 0.15% rice husk, approximated by a two-component Burgers model (The green line is the overall Burgers model response). Other colors highlight the responses of different elements of the Burgers model (viscous dampers and purely elastic springs).

The results of creep and recovery testing with hydrogel and rice-husk additives are shown in Figure 28.

Each element of the analog model corresponds to curves, which, in total, approximate the experimental data.

The features of the rheological behavior of hydrogel with rice husks suggest structural changes in the interpolymer formation under the action of shear loads. The two relaxation times of the viscoelastic medium established during the experiment are due to two types of cross-linking: ionic, due to the bonding of the chromium ion with the polymer, and flocculation, due to the flocculation of dispersed particles by polyacrylamide macromolecules. Ion cross-linking corresponds to a shorter relaxation time, and flocculation corresponds to a longer one, which generally results in a high-quality water shutoff.

**Figure 29:**
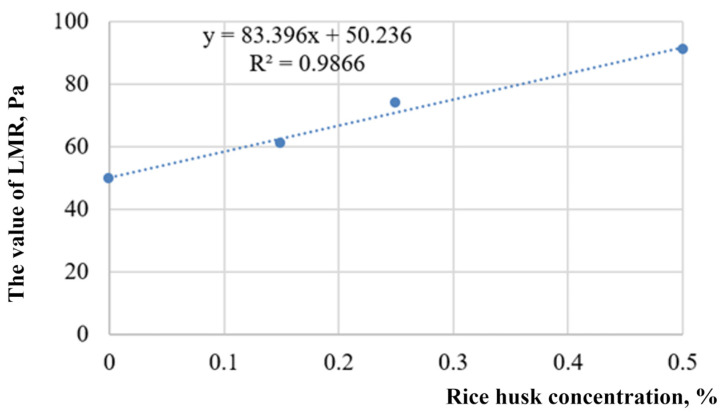
Dependence of the LMR value on the concentration of rice husks in a hybrid hydrogel.

Oscillation experiments allow for one to distinguish a linear measurement range (LMR). In particular, tests showed an increase in LMR, from 50 to 91 Pa, and the maximum shear strain from 104 to 128 Pa, with an increase in the content of dispersed rice husks from 0 to 0.5% (Figure 29).

Detailed rheological studies of hybrid hydrogel with rice husk additives are provided in [146].

The papers [147,148] consider an approach implemented by creating thermotropic compositions, which, under surface conditions, represent low-viscosity aqueous solutions and, directly in the formation under the influence of reservoir temperatures, form cohesive nanoscale structures of the “gel-in-gel” type. A high-temperature nanostructured composition with improved rheological characteristics based on a composition (named MEGA by the authors) consisting of two gel–forming agents—polymer and inorganic—is used for water shutoff in the case of steam cyclic treatment. At a temperature above 70 °C, carbamide hydrolysis occurs in this composition with the formation of ammonia and carbon dioxide, with a gradual increase in the pH of the solution. The aluminum hydroxide gel is produced in the entire volume of the solution when the threshold pH value of 10 is reached.

Thus, the MEGA composition with two gel-forming components based on the system “aluminum salt—cellulose ether—urea—water”, when heated above the lower critical dissolution temperature in the system due to a reversible phase transition, forms a polymer gel. Then, an aluminum hydroxide gel is formed inside it by the mechanism of hydrolytic polycondensation. These gels have viscoplastic and viscoelastic properties because they are capable of elastic restoration of the structure after stress relief. Such gels are characterized by a spatial structure that persists under the impact of shear strain until the value of the latter exceeds the critical value, after which its destruction occurs. The ultimate shear strain of the MEGA composition ranges from 433 to 590 Pa, which is 1.6–2 times higher than the ultimate shear strain of gels based on a single inorganic component. The filtration studies of MEGA gel found that the critical pressure gradient equals 6–14 MPa/m.

A self-generating foam gel composition has been used for gas shutoff in oil wells. Gel in this composition is formed from partially hydrolyzed polyacrylamide and chromium acetate, and the foam is produced by the release of nitrogen from the solutions of salts: sodium nitrite and ammonium chloride. The components of the composition are mixed at the well head, and the time of gelling and gas release can be regulated by varying the concentration of the starting substances, depending on the reservoir temperature. It should be noted that the structural and mechanical properties of the foam gel exceed the properties of pure gel, which makes it possible to solve a rather complex technical problem of gas shutoff in horizontal wells [149].

Another technically difficult task is water shutoff in gas wells. Both traditional hydrogels and hydrocarbon-based gels are used for this purpose. The use of hydrocarbon-based gels looks preferable since gas wells are very sensitive to water-based remedial fluids [150].

### 4.2. Gels with Nanocomponent Additives

The use of nanocomposites has been one of the most significant trends in the development of gel-forming compositions for water shutoff in wells in the last 10 years. Either synthetic polymers or biopolymers are used as base polymers for nanocomposites used for water shutoff. Synthetic polymers are mainly derivatives of acrylamides, including polyacrylamide, partially hydrolyzed polyacrylamide, polyvinyl alcohol, and polyacrylamide–tert–butyl acrylate. They are broadly used in field conditions because they are relatively low cost and easily soluble in water, which makes the technology of their application quite simple. Biopolymers such as xanthan gum and others are fermentation products. Although they are environmentally friendly and form high-viscosity solutions, biopolymers are rarely used in the fields due to their high cost [151].

We will try to highlight in more detail the fundamental advantages of this approach, taking into account the novelty and prospects of using nanoparticles in shutoff compositions.

Various nanocomponent additives are used to increase the success of the use of hydrogel polymer systems for water shutoff and expand the scope of their application in complicated reservoir conditions (temperature, pressure, mineralization of formation water). These include inorganic nanoparticles (such as silicon oxide, titanium oxide, zirconium hydroxide) and organic (cellulose and graphene nanoparticles), which increase the stability and strength of cross-linked gels in reservoir conditions [152].

The authors [153] considered hydrogel systems based on hydrolyzed polyacrylamide, chromium acetate cross-linking, and silica nanoparticles. The study results showed an increase in the gelling time at 90 °C in a hydrogel system with the addition of silica nanoparticles. The gelling time can increase due to the impact of the cluster structure of silica nanoparticles [153,154]. In addition, an up-to-1.5-times decrease of the viscosity of the polymer solution, with an increase of the concentration of nanoparticles, was revealed at a concentration of nanoparticles of 9 wt.%, which simplifies its injection into the formation. Oscillatory studies showed that the addition of nanoparticles increases the storage modulus G′ from 520 Pa (conventional hydrogel) to 26,100 Pa (with added nanoparticles). The results of the determination of viscosity and rheological properties showed that silica nanoparticles not only reduce the viscosity of the polymer system but also increase the strength of the hydrogels formed.

A similar hydrogel with the addition of silica nanoparticles (15–20 nm) was used in the work [155]. The study showed that the gelling time increased at low concentrations of nanoparticles (from 4 h for the initial composition to 9.5 h at a concentration of 0.3 wt.% nanoparticles) but then significantly decreased as the concentration of nanosilicon particles increased (up to 0.5 h at a concentration of 1 wt.% nanoparticles). The increase of gelling time is, thus, attributable to an increase of viscosity, which is associated with a slowdown of the diffusion process. In addition, small particles create obstacles that prevent the effective collision of molecules.

A gel based on colloidal silicon dioxide and various salts (NaCl, KCl, NH_4_Cl, CaCl_2_, NaNO_3_, and Na_2_SO_4_) used as activators was developed in the work [156]. It is noted that silica nanoparticles carry a negative surface charge, due to which they are stabilized in an alkaline solution in which the repulsive forces between equally charged particles prevent them from colliding with each other. Therefore, the initial colloidal system is stable and usually has a high negative value of the zeta potential since it directly determines the repulsive forces between particles in a colloidal solution.

When using a gel-forming system based on nanosilicon to block the inflow of water, it is expected that the gelling process will begin as soon as the colloidal system is destabilized from its initial state. One of the ways to initiate the gelling process is to reduce the repulsive forces between negatively charged silica nanoparticles by introducing opposite ions (cations), the addition of which directly reduces the zeta potential of the system. Consequently, the colloidal system of nanosilicon becomes unstable, and the gelling process begins. At the first stage of gelling, nanoparticles, as a rule, simply come into contact due to a decrease in the repulsive force. However, no covalent bonds are formed, so the system has a low viscosity. As the particles enter into a reaction of condensation-forming covalent bonds, which results in the formation of extended networks represented as aggregates and agglomerates, the system demonstrates an increase of viscosity. A nanosilicon gel is formed at the third stage of gelling, but the strengthening of the gel is still ongoing, although the stability of the system is completely lost.

In general, cross-linkers used for the preparation of cross-linked polymer gels can be represented by both metal ions and organic molecules. Trivalent metal ions cross-link polymers with ionic bonds and organic cross-linkers with covalent bonds. Such cross-linkers as Cr^3+^, Zr^4+^, and Al^3+^ are known as toxic compounds, which is unpractical from an environmental point of view. Moreover, gels cross-linked with metal ions usually have poor thermal stability and a short gelling time at temperatures above ~60–70 °C [157]. Polymer gels cross-linked with organic cross-linkers have better thermal stability and a longer gelling time, even at high temperatures. This is because covalent bonds have a higher binding energy than ionic bonds [158].

**Figure 30:**
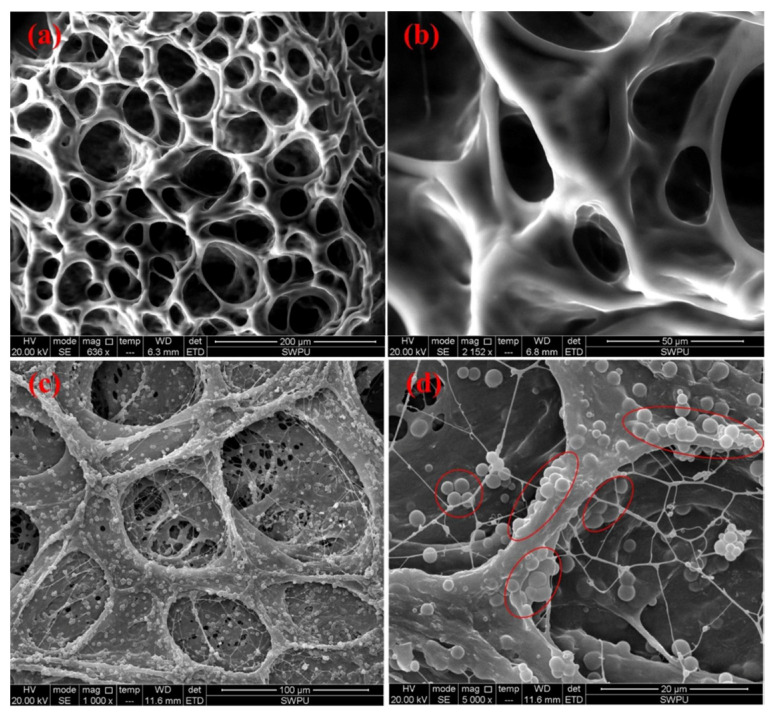
ESEM micrographs of gel samples prepared with different concentrations of silica nanoparticles: (**a**,**b**) Without silica nanoparticles; (**c**,**d**)—0.2 wt.% (Silica nanoparticles aggregations and arrangements are highlighted in red). (Reprinted (adapted) with permission from Liu et al., Ref. [158], Copyright 2017 American Chemical Society).

The reinforcing effect of silica nanoparticles on a gel consisting of polyacrylamide, hydroquinone, and hexamethylenetetramine was studied [158]. The base gel, as well as the gel that contained silica nanoparticles (median size 13 nm) up to 0.3 wt.%, was prepared at 110 °C. When silica nanoparticles were added, the gelling time was noticeably reduced (from 16 to 9 h for a concentration of 0.3 wt.%), and the gel strength increased. Rheological measurements showed that silica nanoparticles significantly increased the elasticity and viscosity of the gel. Thus, the storage modulus G′ increased 6.4 times on average (from 5 to 32 Pa), and the temperature stability of the gel increased from 137.8 °C to 155.5 °C with the addition of silica nanoparticles with a concentration of 0.3 wt.% It should be noted that the content of bound water also increased from 22.5% to 39.9%, which can be explained by the hydrophilicity of silica nanoparticles attributable to the presence of a large number of hydroxyl groups on their surface that can bind water [106]. Improved hydrophilic properties protect the gel from dehydration, which ensures its improved thermal stability (Figure 30).

A modified polymer gel system was obtained in the work [159] using polyacrylamide, polyethylenimine, thiourea, and nanosilicon with a mineralization of 212 mg/L. When nanosilicon particles with an average particle size of 152.1 nm were added to the polymer solution, the gelling time at a temperature of 105 °C was 14 h. The content of bound water in the subject gel system (as well as in the work [158]) increased by 19.5% after the addition of nanosilicon particles. In addition, the residual resistance factor remains high after 30 days at 105 °C (with the addition of 1 wt.% of nanoparticles). The mechanism of hydrogel strengthening by nanoparticles is generalized in this paper with the identification of two reasons. Firstly, hydrophilic nanosilicon can act as a cross-linking agent that generates silanol groups and increases the cross-linking density in a modified gel system. Secondly, the formed silanol groups interacting with segments of polymer chains through hydrogen bonds can significantly reduce the dehydration of the polymer gel system.

Nanocellulose, as a natural and renewable polymer material, is widely used for the preparation of polymer gel systems [160,161,162], ensuring the resistance of nanocomposites to high temperatures and mineralization.

Nanocrystalline cellulose, also known by the name as cellulose nanocrystals, or cellulose nanofibers, is a material with a high strength and is usually extracted from cellulose fibrils by acid hydrolysis [163,164]. Cellulose nanocrystals are one-dimensional particles (diameter 2–20 nm, length 50–300 nm) [164], which, being a renewable natural material, have a unique high crystallinity (crystallinity) in the range of 54–88% [165,166], low density, and excellent mechanical properties [167].

The authors [168] consider the addition of nanocrystalline cellulose to produce a hydrogel (based on an acrylamide monomer, initiator potassium persulfate, and crosslayer N,N′-methylene-bis-acrylamide with high-thixotropic properties). The optimal properties of the gel were achieved at a concentration of nanocrystalline cellulose equal to 10%. However, it was determined that mineralization (NaCl, CaCl_2_) and reservoir temperature have a significant negative effect on properties of the modified hydrogel.

The hydrogel prepared from polyacrylic acid, potassium persulfate, and nanocellulose was considered in the work [169]. The structure of the resulting hydrogel was studied by electron microscopy methods. It was also studied in the deformed state when the mechanical strain is applied. The strength of the modified hydrogel (at different concentrations of nanocellulose) was compared with a gel based on an organic cross layer: N,N′-methylene bis-acrylamide using oscillation methods. The modified hydrogels showed better results in the order of magnitude, which confirms a significant improvement in the mechanical properties of nanocellulose-based waterproofing compounds.

The results of the study of a hydrogel prepared from acrylamide, acrylic acid, ammonium persulfate, and N,N′-methylene bis-acrylamide in the presence of nanocellulose are provided in [170]. It was shown that the addition of nanocellulose (0.2 wt.%) increases the compressive strength of the gel by seven times, as well as the thermal stability and elasticity during shear due to the formation of double cross-linked hydrogels. The combination of acrylamide and acrylic acid monomers, as well as nanocellulose, results in cross-linking due to the interaction of hydrogen bonds with polyacrylamide and polyacrylic acid chains. When aluminum chloride is added to the system a rigid and durable double-cross-linking hydrogel is formed due to its coordination interaction with the carboxyl group, which showed a critical gradient value of 23.73 MPa in filtration experiments, which is an order of magnitude higher than that of a single cross-linking gel (2.87 MPa).

A self-healing hydrogel with double cross-linking was developed in the study [171] for gas shutoff in formations in production wells for implementing enhanced oil-recovery technology based on CO_2_ injection. The gel was synthesized using acrylic acid, heat-treated carboxylated nanocellulose, and Fe^3+^ ions. This gel increases the self-healing properties and withstands a strain of 1.03 MPa and high deformation (1491%). After fracturing, it recovers itself to the original values of up to 98% in terms of strain strength and up to 96% in terms of deformation strength, respectively.

The authors of the paper [172] synthesized pre-crosslinked particles of cellulose-modified hydrogel (nanocellulose-regulated particle-gel) in the process of radical polymerization by the penetration of nanocellulose into the matrix of partially hydrolyzed polyacrylamide.

One of the methods of obtaining strong hydrogels with improved self-healing properties is the preparation of nanocomposite hydrogels by reinforcing polymers with two-dimensional nanofillers, such as graphene, graphene oxide, and boron nitride [173,174,175]. Graphene nanoparticles are also a promising filler for creating heat-resistant hydrogels.

New thermoelastic and self-healing polymer composite hydrogels for high-temperature reservoir conditions were obtained and studied in [175]. The hydrogel was prepared by reinforcing polyacrylamide with a low molecular weight with two-dimensional nanolayer fillers (at concentrations of 0.01–0.1%), including graphene oxide, commercial graphene, and boron nitride. These polymer composite hydrogels were cross-linked using hydroquinone and hexamethylenediamine. An alkali metal salt (for example, potassium chloride) was also added to facilitate the self-healing properties of the hydrogel due to the ionic bond with the amide group of the polyacrylamide chain.

**Figure 31:**
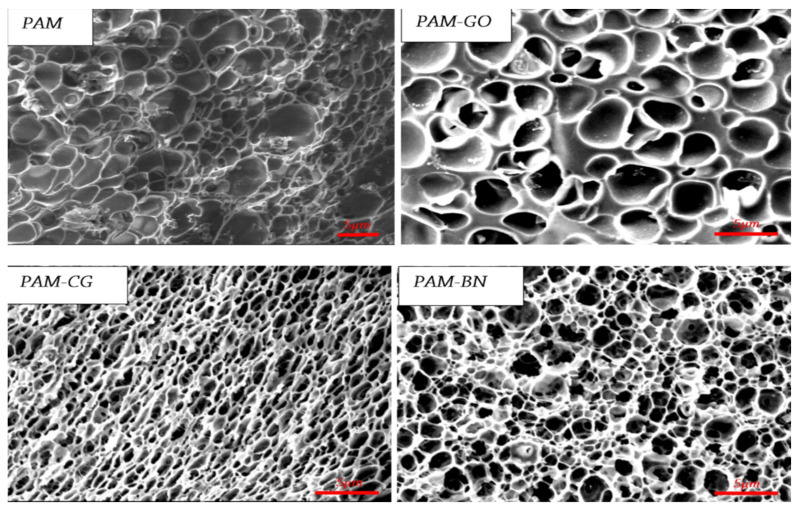
Images (scanning electron microscopy) of composite hydrogels in comparison with conventional hydrogel. (Reprinted/adapted with permission from Michael et al., 2020, Ref. [175]).

Figure 31 shows micrographs of the structure of the base gel and modified gel.

It was determined that the interaction between the matrix of polyacrylamide and graphene oxide occurs through physical cross-linking, while fillers such as commercial graphene and boron nitride interact with polyacrylamide through Van der Waals forces and π–π interactions. The results of oscillatory studies (G′, G″) showed that the inclusion of 2D fillers reinforced the hydrogel matrix despite stretching due to the capture of water molecules inside its structure.

Similar studies of the use of hydrogels for leveling the inflow profile under conditions of steam treatment were carried out in the work [176] where a high-molecular organic polymer was modified by the addition of graphite nanoparticles. Studies showed an increase in the blocking ability of the gel when graphite particles are added.

The paper [173] proposes an approach for the preparation of new elastic graphene oxide—polyacrylamide hydrogels with exceptional mechanical behavior due to the synergistic effect of the interaction of graphene oxide with calcium ions due to the combination of the characteristics of a conventional double-network hydrogel and a hydrogel nanocomposite. Hybrid hydrogel based on graphene oxide and polyacrylamide demonstrated high strength, good elasticity, and super-stretching (up to 1350% of the original length).

New nanocomposites based on zirconium oxide and graphene oxide synthesized in situ by microwave irradiation were used in the work [177] as a cross-linking agent for a water-insulating composition based on polyacrylamide with a low-molecular weight. Nanocomposites were prepared using a simple, cost-effective, environmentally friendly and scalable method of chemical reduction using microwave irradiation. Studies showed that only 0.2 wt.% nanocomposites based on zirconium oxide and graphene oxide formed a highly stable gel at high temperature (150 °C) with improved mechanical properties in case of addition of 4 wt.% of polyacrylamide solution.

### 4.3. Mathematical Modeling of Water Shutoff Operation with Gel-Forming Compositions

Various mathematical models (from statistical to numerical) are used by scientists and engineers of the oil and gas industry to improve the accuracy of predicting the effect of the use of polymer gels and optimize the volume of their injection in water flow restriction technologies [178,179,180,181,182,183]. Treatment modeling allows for the identification of the most significant factors, affecting the results of processing via selecting proper candidate wells and optimizing the process parameters of the treatment.

Statistical models are based on the analysis of the efficiency and process parameters of previously performed treatments.

A large number of studies use regression analysis methods [178,179] to obtain an explicit equation for calculating the predicted parameter (flow rate of oil, water, liquid after water shutoff, watercut). However, such approaches have their drawbacks. For example, a long history of application of the subject technology in specific geological and physical conditions is required because obtained relationships or models do not take into account the physico–chemical and rheological properties of insulating compositions, as well as the structure and structural features of the formation.

The authors of the work [180] use machine-learning algorithm Random Forest method to select candidate wells. The analysis of the results of hydrodynamic modeling, taking into account the water shutoff operations in wells selected by the random forest method, showed greater technological efficiency than the Fuzzy Evaluation Method previously used by the authors.

Other modeling methods can be attributed to analytical ones when solutions of equations derived from basic physical laws are used with significant assumptions and simplifications of the process itself. This approach can be attributed to express methods because it does not require specialized software and is quite simple. However, the considered procedure prevents us from describing with sufficient accuracy the mechanisms of placement of the water shutoff mixture in the formation and during the subsequent operation of the well. Thus, the analytical calculations do not take into account the rheological properties of the water shutoff mixture, which have a significant impact on the process of placement and distribution of the composition in the interlayers of a watered heterogeneous formation. In addition, the depth of penetration is not taken into account explicitly, which prevents the description of the process of the subsequent recovery of the watercut. However, it is possible to use the results of analytical calculations in commercial hydrodynamic simulators (Black Oil) to predict not only the starting indicators, but also the dynamics of operating indicators during the time of the effect [183,184,185,186,187].

Analytical calculations allow for the estimation of the volume distribution of the selective water-insulating mixture *V_i_*, taking into account the thickness *h*, absolute *k*, and phase *k_w_* permeabilities of the layers of a heterogeneous formation:

(1) $$V_{i}=V_{0}\cdot \frac{k_{i}\cdot h_{i}\cdot k_{w}\left(S_{wi}\right)}{\sum_{i=1}^{n}k_{i}\cdot h_{i}\cdot k_{w}\left(S_{wi}\right)},$$

where *V*_0_ is the total volume of the water shutoff composition selected according to the technology of its implementation.

The values of phase permeabilities depend on the water saturation of the subject layers. These parameters can be unloaded from a history-matched hydrodynamic model, or they can be determined analytically.

The radius of penetration *Rsi* of the blocking compound along the interlayers is calculated using the formula:

(2) $$Rsi=\sqrt{rw^{2}+\frac{V_{i}}{m_{i}\cdot \pi \cdot h_{i}\cdot \left(1-Sowcr-Swcr\right)}},$$

where: $m_{i}$—porosity of the *i*-th layer, u.f.; Rsi—radius of the formed water shutoff screen, m; rw—radius of the well, m; Sowcr—residual oil saturation, u.f.; Swcr—connate water saturation, u.f.

According to the Hawkins formula [188] the skin factor is calculated for each layer *S_i_*, taking into account the residual resistance factor of the shutoff composition (*Frr*):

(3) $$S_{i}=\ln\frac{Rsi}{rw}\cdot \left(Frr-1\right).$$

The corresponding water and oil productivity indexes, the startup flow rate of the well, and the watercut can be determined based on the calculated values of the skin factor for each layer. They can also be loaded into a hydrodynamic model, and predictive calculations of well operation indicators will be performed.

There are a large number of software products that allow the modeling of the implementation of various physico–chemical-enhanced oil recovery methods within which there is the possibility to simulate the use of cross-linked gels. These software products include three-dimensional multiphase multicomponent hydrodynamic simulator UTCHEM (created by Shell, London, UK, currently supported by scientists from the University of Texas (UT Austin)), STARS (Computer Modeling Group (CMG), Calgary, AB, Canada), ECLIPSE 300 (Schlumberger, Houston, TX, USA), SCORPIO (Simulator for Chemical Oil Recovery and Polymer Injection from AEA Petroleum Services), PC-GEL (a joint project of the Illinois Institute of Technology Research Institute (IITRI, Chicago, IL, USA) and the US National Institute for Petroleum and Energy Research (NIPER), POL-GEL (Institute of Petroleum Exploration and Development (RIPED)), PUMAFLOW (Beicip–Franlab and the French Institute of Petroleum (IFP)), VIP (Landmark, Houston, TX, USA and Halliburton, Houston, TX, USA), REVEAL (Petroleum Experts, Edinburgh, UK), IORCoreSim (University of Stavanger (UIs), Norwegian Research Center (NORCE), and the Norwegian Institute of Energy Technology (IFE)) [189].

For example, in the works [190,191], the modeling of the injection of gels for water shutoff is performed in the CMG STARS software package. The kinetics of gelling were described in terms of reaction rate constants, where gel adsorption in the formation was considered.

The works [192,193] consider the use of the POL–GEL simulator, which is a simulator with 3-dimensional, 3-phase (oil/gas/water), 9-component (oil/gas/water/polymer/cross-linker/gel/monovalent ions/divalent ions/additional sensitive component). The simulator can be used to simulate all types of gel-forming polymers that have appeared recently. The simulator takes into account the main mechanisms, physico–chemical phenomena, and factors influencing the use of cross-linked and non-cross-linked polymer systems. The model considers the processes of gel formation, reduced permeability, and changes in fluidity.

The paper [194] analyzes articles on the methods of modeling the use of gel systems in the processes of limiting water inflow for various technologies, including those implemented in the simulators discussed above. It is noted that in situ gel modeling is quite a difficult task since the viscosity and flow regime in the system vary greatly before and after gelling. In addition, the system has both liquid and solid properties during the cross-linking process and after gel formation. The author divides the evolution of the gel composition injected into the reservoir into three stages: non-cross-linked polymer solution, gel formation, and cross-linked gel. In each of the stages considered, the gel composition will have different physicochemical and, especially, rheological properties that require different approaches to modeling.

However, this approach prevents us from fully taking into account all the features of gel injection, gel formation, and subsequent behavior of the blocking screen since commercial simulators implement a more general approach for modeling chemical EOR methods.

The last group includes separate mathematical models that require the use of numerical methods of solution and implementation in program code [182,194,195]. In general, the task of mathematically modeling the process of injection and placement of water shutoff compositions in the bottom-hole zone of the formation to block the watered intervals of the formation, oil, and water inflow conformance control. Redistribution of filtration flows in the reservoir in the well area has many options for meaningful formulation and solutions determined by a significant number of physicochemical processes occurring in the near-well zone of the formation at filtration of reservoir fluids and water-insulating compositions in it, including the gelling of the polymer composition used and the subsequent interaction of the formed gel with reservoir fluids under dynamic conditions [195,196].

**Figure 32:**
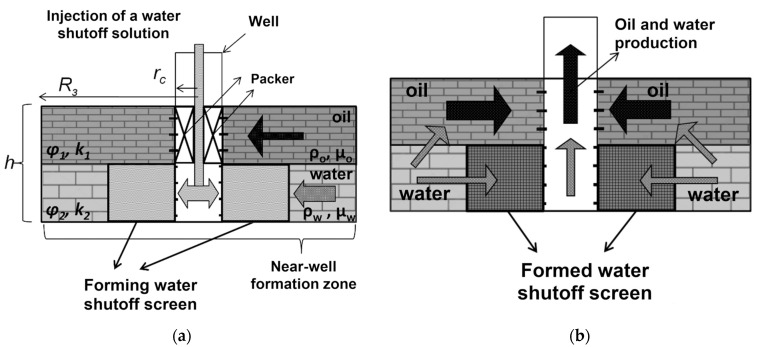
Simplified scheme of the near-well zone of the watered oil formation and the water shutoff combined with chemical–technological process: (**a**) Injection of a water shutoff solution; (**b**) The physico–chemical process of gelling of the solution and filtration of oil and water, taking into account vertical flows. r_c_ is the radius of the well, m; R_3_ is the radius of the near–well zone of the formation, m; h is the thickness of the formation, m; φ is porosity, u.f.; k is permeability, µm^2^; ρ_w_, ρ_o_ is the density of water and oil, respectively, kg/m^3^; µ_w_, µ_o_ is the viscosity of water and oil, respectively, mPa∙s. (Reprinted/adapted with permission from Meshalkin et al., 2021, Ref. [195]).

In the paper [195], the authors considered a combined chemical and technological process of water shutoff of a watered oil formation in the form of a combination of the following processes (Figure 32): hydrodynamic process of pumping an organo–mineral solution, physico–chemical process of gelling an organo-mineral solution, and the process of two-phase filtration of oil and water, taking into account vertical flows.

A number of prerequisites were adopted to substantiate the methodology of mathematical modeling of water shutoff processes using cross-linked polymers and liquid filtration processes in the near-well zone of the formation: a model of two-phase filtration (water and oil phases) was used, which will allow the consideration of the impact of phase viscosity on the injection of a gel-forming agent and performing predictive calculations of changes of well operation parameters (watercut, oil flow rates) after water shutoff; a layered-inhomogeneous formation with several layers with different reservoir properties is considered; vertical anisotropy of permeability is taken into account to calculate vertical filtration flows between layers of different permeability to quantify changes in the direction of oil and water flows in the near-well zone of the formation after water shutoff; and the numerical model is implemented using a three-dimensional calculated grid, which is necessary to account for the heterogeneity of the reservoir permeability. However, the mathematical model was developed without taking into account the compressibility of the fluids under consideration and the elasticity of the porous medium of the formation; the gravitational component of the pressure gradient field, which is due to the short duration of the injection of process fluids and small values of pressure gradients created by gravitational forces in comparison with existing filtration gradients in the bottom-hole area during the entire water shutoff process; and capillary pressures, since the existing pressure gradients in the bottom-hole area during injection of process fluids and subsequent operation of the well after the job significantly exceeded them.

**Figure 33:**
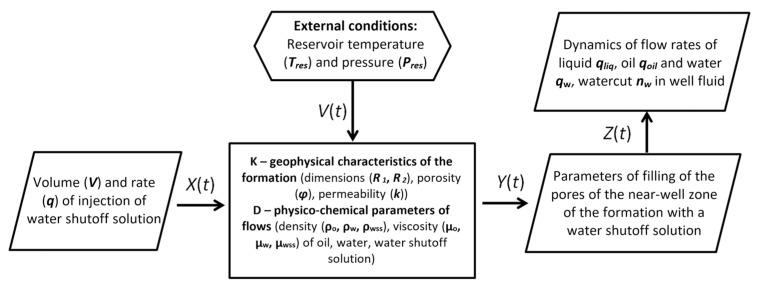
Simplified block diagram of a mathematical model of the chemical and technological process of water shutoff of an oil reservoir. *X(t)*—input parameters (process characteristics); *Y(t)*—output parameters (calculation results); *Z(t)*—calculation functions of additional parameters characterizing the effectiveness of the operation. (Reprinted/adapted with permission from Meshalkin et al., 2021, Ref. [195]).

A generalized block diagram of the mathematical model is shown in Figure 33.

The mathematical model of the filtration process consists of the following basic equations:

1. Flow continuity equations (for the water and oil phase) [78]:

(4) $$\frac{d(\varphi \cdot \rho_{\alpha }\cdot S_{\alpha })}{dt}=-div\left({\rho_{\alpha }\cdot u}_{\alpha }\right)+q_{\alpha },$$

where *α*—the index corresponding to a certain phase: *w* is water, *o* is oil; *ρ_α_* is the density of the phase *α*, kg/m^3^; φ—the coefficient of open porosity, u.f.; *t* is the process time, s; *S_α_* is the saturation of the phase *α*, u.f.; *u_α_*—phase filtration velocity, m/s; *q_α_*—mass density of the drain or source of phase *α*, kg/m^3^ s^−1^:

2. Darcy’s linear filtration law:

(5) $$u_{\alpha }=-\frac{k_{r\alpha }}{\mu_{\alpha }}\cdot K\cdot (\nabla P-\rho_{\alpha }\cdot g\cdot \nabla z),$$

where *k_rα_* is the relative phase permeability, u.f.; *µ_α_* is the coefficient of dynamic viscosity, mPa∙s; *K* is the tensor of the coefficient of absolute permeability of a porous medium, µm^2^; *P* is pressure, Pa; *g* is the acceleration of gravity, m/s^2^; *z* is the vertical coordinate, m.

3. Equation of normalization of saturation of pore space:

(6) $$S_{w}+S_{o}=1.$$

For the numerical solution of a well-founded system of equations of the mathematical model (4, 5, 6), the implicit pressure explicit saturation method IMPES (implicit pressure explicit saturations) is used [197,198], which implies a sequential solution for pressure using the implicit method of the equation and the explicit method of the equation for saturation. The discretization of differential equations is performed using the finite difference method. The presented mathematical model and the numerical scheme of its solution were implemented in the program code in the MATLAB programming language.

The process of injection of the gel-forming composition into the bottom-hole zone of the formation is described by the continuity equation for the active component of the mixture:

(7) $$\varphi \cdot \frac{d{(C\cdot S}_{w})}{dt}=-div\left(u_{t}\cdot f_{w}\cdot C\right)+q_{vw}\cdot C,$$

where *C* is the concentration of the component of the water shutoff solution in the aqueous phase, u.f.

The physical and chemical model of the gelling process in this work consists of reducing the phase permeability in the model cells filled with a water shutoff solution according to the following dependence:

(8) $$k_{\alpha i,j,k}^{\prime }=\frac{k_{\alpha i,j,k}}{1+(R_{\alpha }-1)\cdot C_{i,j,k}},$$

where $k_{i,j,k}^{\prime }$ is the permeability of the cell (*i*, *j*, *k*) after the gelling of the water shutoff composition, µm^2^; $k_{i,j,k}$;—the initial permeability of the cell (*i*, *j*, *k*), µm^2^; *R_α_* is the residual resistance factor for the phase *α,* d. units; $C_{i,j,k}$—the concentration of the water-insulating solution, u.f.

The residual resistance factor R_α_ is determined by the results of filtration studies on core samples in the laboratory.

The validation stage (confirmation of adequacy) of the proposed computer model consisted of performing predictive calculations of the efficiency of water shutoff operations using a gel-forming composition (the authors’ technology) for a real object—well C1 of the oil field M. Pilot field tests of the subject technology were conducted in this well. The real geological and physical characteristics of the object and technological parameters of well processing were incorporated into the computer model.

**Figure 34:**
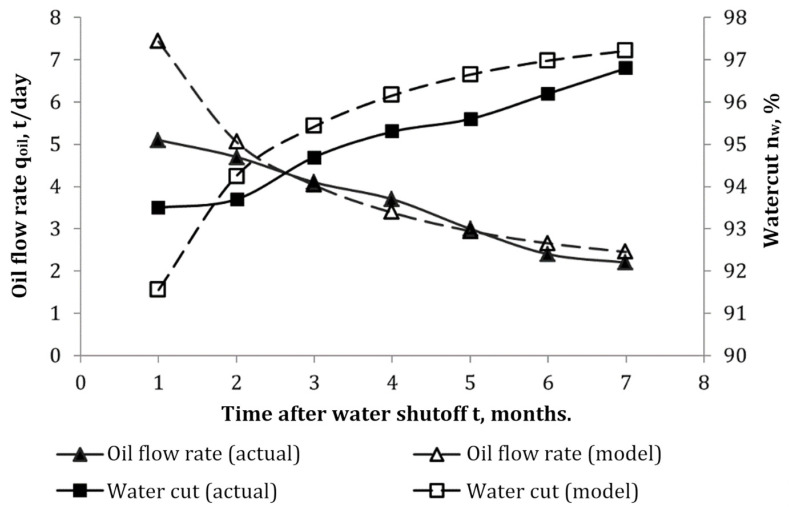
Comparison of actual and calculated values of oil flow rate and watercut after water shutoff. (Reprinted/adapted with permission from Meshalkin et al., 2021, Ref. [195]).

Figure 34 shows the graphs confirming the adequacy of the model; the dynamics of the actual and forecast parameters of the operation of the well C1 (watercut of well fluid and oil-flow rate) after water shutoff works are carried out. The average absolute error (MAE, Mean Absolute Error) was calculated to assess the forecast quality metric: its value was 0.5 t/day for oil flow rate and 0.9% for oil flow rate.

The approach considered above provides more flexibility than commercial simulators for addressing the issues of modeling of water shutoff operations, directly taking into account the results of physical and chemical, rheological and filtration studies of compositions and allows for the use of the obtained dependences of the properties of the polymer solution and gel for specific conditions (for example, the dependence of the residual resistance factor on the pressure gradient).

Summarizing this section, it can be noted that gels for water and gas shutoff in wells are based on the same polymers and inorganic compounds as flow-diversion gels. The difference lies in the fact that the hydrodynamic conditions under which these gels are operated in producing wells are more severe. Therefore, the concentrations of the active base are higher, and the values of the structural and mechanical characteristics of water isolation screens are higher. Hybrid organo–inorganic gel screens are now being used to provide selective water isolation in fractured reservoirs based on fluid type and permeability heterogeneity. Microcomposite and nanocomposite materials are used to increase thermal stability and strength characteristics. Considering that the success rate of water shutoff operations in the oil industry is low, mathematical modeling has recently been carried out to increase its effectiveness. This has led to a more rigorous determination of the volume of gel injection, increased technological and economic efficiency of the process, and prioritization of wells for treatment on large fields.

## 5. Gels for EOR

**Figure 35:**
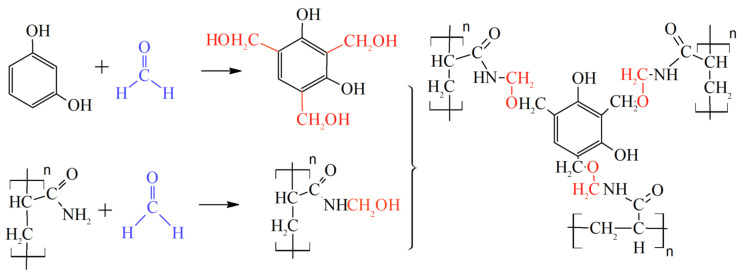
The reaction scheme for the polymer-gel system formation.

The use of acrylamide polymers in polymer flooding technology is one of the most reliable and widespread methods of increasing oil recovery for fields with viscous oil [199]. However, polymer flooding in its pure form is not a technology with the use of hydrogels, but its modifications can already be safely attributed to the use of hydrogels for enhanced oil recovery. In particular, I. A. Shvetsov at the end of the 1980s developed, successfully tested, and implemented the technology of hi-vis pill polymer flooding at the Kalamkas field in Kazakhstan [200]. This field is characterized not only by high oil viscosity but also by high layer-by-layer permeability heterogeneity. In this regard, at the first stage, the conformance control was performed using viscoelastic polyacrylamide rims with a chrome cross-linker, and only then the linear polyacrylamide slug was injected to align the mobility of displaced and displacing agents. The technology turned out to be very effective, which inspired the authors to develop a software product for modeling this process since the known programs were applicable only for pure polymer flooding [200,201]. Another modification of polymer flooding is the gel polymer flooding technology implemented at the Severnye Buzachi field (Kazakhstan) [201,202,203]. This deposit has a very high oil viscosity (400–500 mPa·s) and a very large permeability heterogeneity. An international group of specialists from China, Kazakhstan, and Russia has introduced a new modification that is used for the development of the field to this day. Therefore, at the first stage, a water-swellable phenolaldehyde resin is injected to equalize the injectivity profile, which can be safely attributed to gel technologies. At the second stage, polyacrylamide is injected with a complex organic cross-linker: a mixture of formalin and resorcinol. As a result, a so-called rarely cross-linked polymer is formed, which has viscoelastic properties and is easily filtered in a porous medium. Such a technique not only provides the displacing agent with valuable rheological properties but also allows for the saving of an expensive polymer. The reaction scheme for the formation of the polymer-gel system is shown in Figure 35.

Polymer flooding by the injection of colloidal-dispersed gels (CDG, Tiorco, Inc.), which are low-concentrated polyacrylamide solutions with a concentration of 0.03–0.07%, cross-linked with aluminum citrate, occupies an intermediate position between the actual flooding and conformance control by the volume of injection. However, guided by the mechanism of oil displacement, we still use this technology attributed to the modification of polymer flooding [204,205].

When it comes to the application of gels for enhanced oil recovery, it should be noted that there are only a few projects in global practice, while polymer flooding is one of the most common methods. This is because the reservoir of geological conditions and fluid properties that meet the criteria for the applicability of polymer flooding are much more common. For polymer-gel flooding, the reservoir should have viscous oil (more than 100 mPa·s) and be permeability heterogeneous, meaning it is a reservoir where the alignment of mobility of displacing and displaced agents is insufficient. Permeability heterogeneity should be equalized as well. However, for objects like the West Kazakhstan fields, the option of gel-polymer flooding is promising and economically justified.

## 6. Gels for Acid Stimulation of Wells

One of the most popular methods of stimulation of wells in carbonate reservoirs is acid treatment; gels of various nature find their application in this technology. Taking into account the permeability heterogeneity of the bottom-hole zone of the formation, combined well treatment technologies assume the injection of a gel-forming agent at the first stage to seal fractures and highly permeable intervals. After the flow-regulating slug, a stimulating acid slug is pumped for treatment of low-permeable intervals. These types of combined effects include directed acid treatment using hydrolyzed polyacrylonitrile or the KARFAS reagent based on an inorganic gel [206].

The injection of self-diverting acids with viscoelastic surfactants has been the most effective and rapidly developing type of acid treatment using gels over the past 20 years [207]. The advantage of such treatment is the ability of the acid composition to accumulate viscoelastic properties during the treatment of the well, and then to collapse when the inflow is triggered. There are many types of surfactants capable of forming viscoelastic solutions. These are zwitterionic amphoteric compounds, such as oleylamidopropylbetaine; cationic surfactants, such as erucyl-bis-(2-hydroxyethyl)chloride; anionic surfactants, such as sulfosuccinates; amino oxides and amidoaminoxides, such as dimethylaminopropylamidooxide of tallic acid; and ethoxylated fatty amines. The mechanism of action of self-diverting acid compositions is based on their ability to multiply the viscosity during reaction with carbonate rock. The pH of the solution increases in the water-saturated intervals of the formation when the acid composition reacts with carbonate rock, and the formation of Ca^2+^ and Mg^2+^ ions causes surfactant molecules to form long rod-like micelles, which leads to high viscosity. As the viscosity increases, the composition directs the next portion of acid to the low-permeable oil-saturated intervals of the formation [208].

Acid compositions with zwitterion-amphoteric surfactants have become the most commonly applied in oilfield operations [209,210,211]. The rheological properties of an acid viscoelastic gel based on oleylamidopropylbetaine and anionic surfactant were studied in detail in [212,213]. It was shown that the rheological behavior of the gel under study is well-described by the Maxwell model with one relaxation time according to the data of oscillatory rheometry, which indicates the interweaving of long cylindrical micelles and the formation of a mesh of meshes by them, which can be destroyed and restored under the impact of external conditions. The use of self-diverting acid compositions allows for the minimization of the volume of acid injection for the formation of dominant wormholes, which facilitates the withdrawal of the well to a potential flow rate due to the ease of the removal of surfactant-based gels during filtration of both oil and water [214].

When it comes to the application of gels for acid well stimulation, it should be noted that the number of such treatments is increasing year by year, and the injection method is determined by geological conditions. For matrix acid stimulation, diverting acid systems are undoubtedly the most effective, while for fractured reservoirs, directional acid treatment is used where a strong gel is first injected to block the fractures, followed by regular or diverting acid composition to increase the conductivity of the pore matrix.

## 7. Gels for Well Drilling

Water-based drill muds are the most common type of drill muds that have been widely used in the oil industry for more than 150 years [215].

Drilling muds are usually divided into three main types: water-based muds (WBM), hydrocarbon-based muds (HBM), and synthetic-based muds (SBM) [215].

At the moment, the actual problems of drilling oil and gas wells are:

- Loss of circulation of the drilling flushing fluid [216,217,218,219].
- Uncontrolled inflow of formation fluid into the well [220].
- Regulation of the density of the drilling flushing fluid [215,221,222].
- Swelling of clay rocks [223,224].
- Thermal stability of the drilling flushing fluid [215,217,223].
- Resistance of the drilling flushing fluid to aggressive environments.

The drilling practice has shown that one of the promising methods for obtaining drill muds is the use of gel technologies, which are understood as obtaining materials with certain chemical and physico–mechanical properties due to the formation of sol and its transfer to gel during condensation, as well as the formation of a polymer spatial grid [225,226].

Much attention is paid to the loss of circulation of the drilling flushing fluid. This problem is widespread due to the diversity and heterogeneity of composing rocks with fractures and cavities [216,217,218,219].

In particular, absorbing and pressurized formations are drilled without complications using gel-forming drill muds based on nepheline concentrate with mineral and dry organic acid additives. The temporary blocking of formations is also achieved using viscoelastic mixtures based on water-soluble cellulose esters and lignosulfonates and combined silicate reagents—regulators of structural and rheological properties of drill muds [227].

Cross-linked polymer-gel systems are among the most effective among various materials for controlling drill mud losses, since they can seal fractures of various openings, strengthen the borehole, and provide solutions to absorption problems in extreme drilling conditions [228,229]. To combat the absorption of drilling mud in fractured high-temperature formations, a gel based on polyacrylamide–polyethylenimine has proven itself well. It has been shown that this cross-linked gel effectively clogs multiple and complex fractures near the borehole [230].

**Figure 36:**
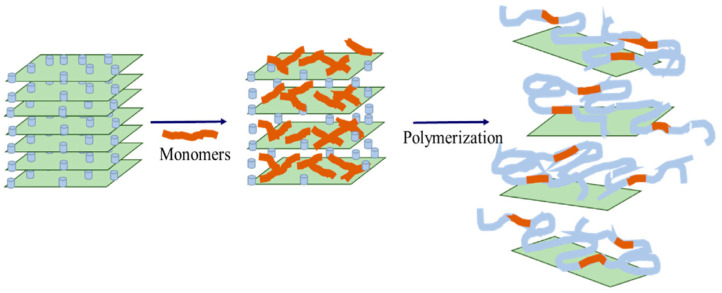
Schematic diagram of in situ intercalated polymerization [232].

New copolymers are synthesized that reduce the cost of the used PAM. The creation of intercalated polymers [231,232], the synthesis of which is shown in Figure 36, is of interest when used in drilling. This type of polymer can be used with a cross-linker.

**Figure 37:**
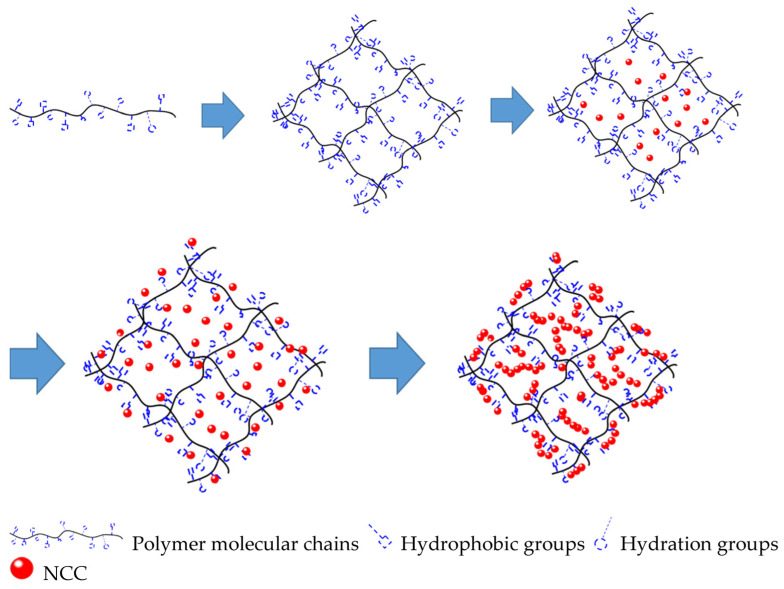
Schematic diagram of PAMSM–CaCO_3_ reaction [217].

A polyacrylamide gel is proposed to prevent and eliminate the loss of drill mud circulation. The traditionally used polyethylenimine is replaced in polyacrylamide gel by functionalized silicon dioxide. In addition, nanosilicon strengthens the gel structure and improves its stability [233]. The works [217,234] show the synthesis of a nanoscale plugging gel to stabilize the borehole during drilling. Synthesized polymer nanospheres of a double cross-linked structure using monomers (styrene, acrylamide, 2–acrylamide-2-methylpropanesulfonic acid and dimethyldiallylammonium chloride) have a complex of necessary plugging properties in the temperature regime of 150–200 °C [234]. Figure 37 shows the scheme of interaction of the synthesized polymer with hydrophobic groups and nanoparticles of calcium carbonate (nano-calcium carbonate, NCC) proposed by the authors [217], which also ensures the stability of systems at high temperatures and mineralization.

Many authors devote their works to the inclusion of nanoparticles and their stabilization in drill muds [217,224,233,234]. It should be noted that these solutions at the moment, for the most part, have been studied only in laboratories. There is a task of reducing the cost of these reagents for use in large volumes for well drilling.

The paper [235] demonstrates the advantages of acid and alkaline silicate gels when drilling wells. In particular, their ability to prevent collapses was proven. Factors affecting the effect of a silicate drill mud on the walls of the well, and the protection of the productive reservoir, were studied. In addition, it was determined that silicate drill muds protect drill pipes and casing strings from corrosion.

Drill muds based on gels containing modified starch, aluminum sulfate, and sodium silicate make it possible to obtain a stable gel-like system of the required density without a solid phase, which prevents peptization of drilled clay rocks and ensures their effective removal from the flushing fluid [236]. The use of this drill mud allows for drilling wells in clay rocks without complications.

The use of Premium-Gel drill mud based on starch and calcium, and potassium and magnesium chloride salts, as well as caustic soda, ensures the stability of active and softened clay deposits, high-quality cleaning of the borehole, and the preservation of the productive reservoir [237]. In addition, a specialized drill mud called “hydrogel” based on inorganic components is used for drilling horizontal holes in clay-containing reservoirs [238]; a similar approach to well drilling in reservoirs with active clays is described in [239].

Cellulose esters are also used in drill muds. Polymer-gel clay drill mud based on polyethylene cellulose and polyanionic cellulose was successfully tested when drilling unstable rocks for drilling horizontal directional wells under conditions of abnormally high-reservoir pressures [240]. Hydroxyethyl cellulose and the possibility of cross-linking this polymer with divalent salts contained in reservoir water are considered in [220].

**Figure 38:**
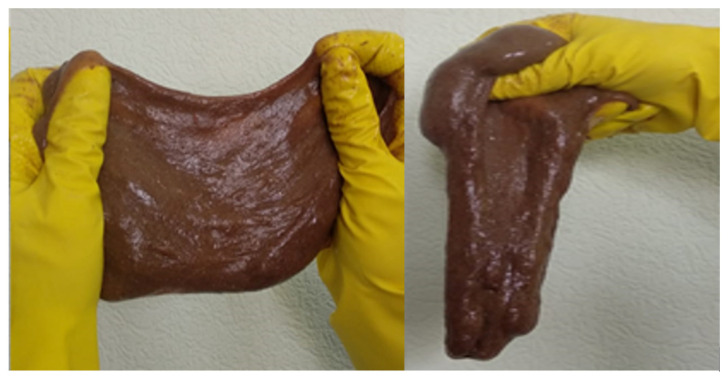
Photo of the grouting material.

The use of hydrogels filled with mineral fibrous-dispersed materials seems very promising [241]. In particular, a grouting compound based on a low-molecular partially hydrolyzed polyacrylamide cross-linked with organic cross-linkers (a mixture of resorcinol and paraform), reinforced with polypropylene fiber, 6-mm long and chrysotile (3MgO∙2SiO_2_∙2H_2_O), which can split into microfibers 0.5-microns thick, limiting disastrous lost circulation during drilling of the Serpukhov horizon at the fields of the Volga–Ural region [25,236]. The appearance of the grouting material selected during testing at one of the wells is shown in Figure 38.

It should be noted that in modern drilling technology, the use of hydrogels is not as common as the use of polymer–clay drilling fluids and inverse emulsions. The main and relevant directions in the application of gel technologies are the search for economically advantageous systems based on polymers, dispersed particles, and additional components, allowing for the creation of a highly structured system. Gels developed to date using various nanoparticles as fillers are mainly at the laboratory development stage and have a low level of implementation in wells, indicating the need to continue creating new fillers and studying the mechanisms of their interaction with polymers.

## 8. Well-Killing Gels

Density is an important property of the well-killing fluid. In general, without any complications, water or an aqueous solution of salts of a certain density can act as a silencing fluid. The variety and relevance of the development of new compositions of killing fluids is attributable to the anomalous value of the reservoir pressure coefficient; the presence of aggressive media; the mud losses in fractures and highly permeable intervals; and the low-viscosity well killing fluids with high penetrating ability, which worsen the reservoir properties of the productive formations.

The following solutions can be used for the listed complications:

- Use of hydrocarbon base (reverse emulsion, thickened oil).
- Introduction of special modifying additives to the well killing fluid.
- Creation of a colloidal system from the well killing fluid (gel, foam, emulsion, etc.).
- The use of blocking compounds in combination with a well killing fluid.

The use of hydrogel as a well killing fluid can solve many of the complications. In this case, there is a problem associated with the subsequent development of the well. This fact determines the main advantage of using natural polymers (polysaccharides) over synthetic ones, which is the possibility of a complete-and-easy destruction of the system. High susceptibility to biodegradation, which in conditions favorable for the development of destructive microorganisms can take several days, significantly reduces the effectiveness of their use. The introduction of biocides to process fluids is a widespread practice and successfully solves the problem of biodegradation, including biodegradation of biopolymer solutions [242].

It is possible to use xanthan as a thickening component of a water-based killing fluid since this polymer can provide it with high performance [242].

It is known that various thickened well-killing fluids are used to prevent the penetration of salt-based well-killing fluids to the formation as they have increased viscosity and a low coefficient of filtration into the formation. The use of thickened well-killing fluids is associated with low-reservoir pressure when the reservoir pressure is lower than hydrostatic. E. N. Kozlov, in [243], proposes well killing and flushing polysaccharide fluids on a water or water-alcohol basis, which constitute gels based on modified guars.

The main parameter of a killing fluid is the density, the value of which should balance the pressure inside the well-formation system. The possibility of destruction and the absence of colmatation of the bottomhole area with degradation of reservoir properties of a productive formation. It is possible to create drawdown and ensure filtration of the killing fluid into the formation when the reservoir pressure anomaly coefficient is lower than the corresponding value of the density of the well-killing fluid column. The solution to this problem can be the use of cross-linked foam systems and blocking compounds. The cross-linked foam system is a pre-foamed polymer solution cross-linked on the flow. The viscosity of such a system is higher than the viscosity of the initial polymer solution and the density can reach a value of less than 500 kg/m^3^. The blocking composition is a cross-linked gel with the added colmatating filler that can be retained at the well bottom without filtration into the absorbing layer.

**Figure 39:**
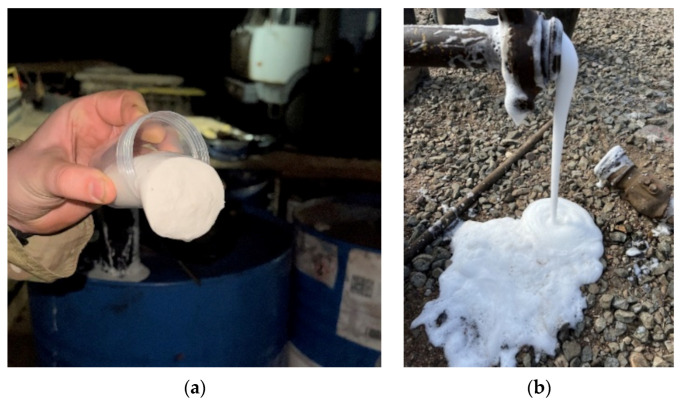
Photo of killing fluid based on guar and xanthan: (**a**) Blocking compound; (**b**) Cross-linked foam.

Gubkin University developed a technology for killing gas wells with disastrous lost circulation in a carbonate reservoir with a reservoir pressure anomaly coefficient below 0.5. The technology comprises the use of a blocking compound based on cross-linked guar with microcalcite filler and cross-linked foam based on guar and xanthan as a killing fluid with a density of about 500 kg/m^3^. The appearance of the blocking compound and the cross-linked foam selected during testing in one of the wells are shown in Figure 39.

There are alternative well-killing fluids in the oil industry for killing low-pressure formations, including foam liquid, oil-based emulsion liquid, and well-killing fluid with a density-reducing agent. However, the density of these alternative fluids for well killing is generally above 800 kg/m^3^. A formula of nitrogen foaming liquid for well killing was developed in [244], where a xanthan gum was chosen as a foam stabilizer since it can thicken the liquid phase and reduce the rate of destruction. In addition, gelatin was added to the composition since it can form a stable coacervate with xanthan gum.

A relatively new direction in the development of silencing fluids is the use of brines based on formic acid salts sodium and potassium formates, which allow you to adjust the density in a very large range. Formates have undoubted advantages over commonly used salts. As strong water-structured antioxidants, they allow the thermal stability of polysaccharides to increase, such as xanthan gum, starches, and cellulose derivatives, especially during long-term operation [243].

Xiong Ying et al., in [245], proposes the use of a gel-blocking system to solve the problems of losing a killing fluid in low-pressure wells; low technical strength of existing gel blocking pills for temporary blocking during well killing; difficult-to-control cross-linking time; embrittlement of gel; and the complexity of destruction of some gel-blocking pills. A mixture of esterified plant gum galactomannan, surfactant polyoxyethylene ether isooctanol, and an oil phase was used as a thickener. A complex of inorganic salts containing a long-chain polyhydroxy alcohol was used as a cross-linking agent, and the concentration of long-chain polyhydroxy alcohol significantly exceeded the theoretical amount required for metal-ion binding. A mixture of polyhydroxy alcohol with a small amount of weak acid was used as a cross-linking regulator. A mixture of sodium thiosulfate and a long-chain quaternary ammonium salt of a surfactant was used as a stabilizer.

Martyushev D.A. et al. in [246] propose a new viscoelastic gel with an adjustable fracture time for oil well killing. Guar and xanthan gum are the basis of the developed VEG A for oil-well killing. A borate cross-linker is the complexing reagent. Guar gum is a gelling, stabilizing, and thickening agent. Xanthan gum is used as a gelling agent and plasticizer. It provides the subject liquid with high rheological and pseudoplastic properties. This simplifies the pumping of the composition into the well with a sufficiently high relative viscosity. In addition, xanthan gum provides well-killing fluids with an increased thermal stability. It also ensures stable rheological and pseudoplastic properties at high temperatures. Due to this, the subject composition can be used in deep formations with temperatures up to 90 °C, unlike known compositions that can be used at temperatures no higher than 50 °C.

Weighting additives are added to the well-killing fluid for killing wells with a reservoir pressure anomaly coefficient exceeding 1.2. The need for a highly viscous gel structure in the well-killing fluid in this case is determined by the retention capacity of the weighting particles in suspension.

Mardashov D. V. et al., in their work [247], conclude that compositions based on caesium formate are the most effective well-killing fluids for AHRP, but their high cost prevent the use of these well-killing fluids on a commercial scale. Compositions based on potash (potassium carbonate) demonstrate an optimal efficiency in conditions of abnormally high reservoir pressures.

Well-killing fluids with a high density of 1.80, 1.70, and 1.60 g/cm^3^ were developed in the study [248] to ensure the safety and efficiency of well-killing operations during oil and gas development based on the requirements of high density, low corrosion, suitable pH and density properties, absence of solid particles, low damage, and biological toxicity.

Thus, it can be concluded that hydrogels based on polymers such as xanthan and guar gum are actively used as well-killing fluids.

In the last 15 years, fractured well killing in low-permeability hydrophilic reservoirs has been carried out using blocking fluids that provide absorption control [249,250,251,252,253,254]. Before the main killing fluid is injected using this technology, a block composition based on xanthan gel is pumped in, in which particles of microcalcite or halite are suspended. The optimal fractional composition of microcalcite is calculated using the theory of ideal packing and the Kaeuffer criterion [255] for different types and sizes of proppant, taking into account their translucency. This approach allows for easy wellbore cleanup after workover by preventing absorption of water solutions and preserving oil phase permeability.

It should be noted that the trend towards modernizing and developing new compositions based on biopolymers for well-killing fluids is driven by the possibility of complete destruction and removal of these fluids from the wellbore. Due to their widespread use and characteristics, xanthan and guar gum are receiving the most attention in terms of hydrogels.

The depletion of oil and gas reservoirs leads to a decrease in reservoir pressure and, as a result, the absorption of fluids in the wellbore, creating repression on the reservoir. Therefore, scientists are focusing on developing well-killing fluids with a density less than 1000 kg/m^3^. In such cases, foaming the well-killing fluid and subsequently stabilizing this system using hydrogels is often considered. Another promising direction for using hydrogels in well killing is their ability to form sedimentation-stable suspensions with particles of microcalcite and halite. Injecting such blocking fluids into the perforation interval of wells ensures the preservation of the reservoir from the penetration of well-killing fluids, which is especially relevant in water-sensitive hydrophilic collectors.

## 9. Conclusions

The most significant trends in the development of the use of gel technologies in oil production processes can be highlighted in the conclusion of the review:

- In general, this is a relevant technological area. It is developing rapidly in all the mentioned processes.
- A wide variety of gel compositions allows for the selection of the optimal composition for any geological and physical conditions of wells for hydraulic fracturing. The use of viscoelastic surfactants currently is the most promising direction of gel studies for this process. With a minimum content of components, they have a good sand-bearing capacity and do not have a damaging effect on the productive layer and the proppant packing.
- The tendency to increase the depth of well treatment with gels with the lower gelling concentration limit should be noted in flow-diversion technologies, which makes it possible to pump large volumes of flow diverters and significantly increase the sweep efficiency. In case of conformance control in wells along the section, the modern development of this technology has come to the injection of low-volume rigid gel-forming and gel-dispersed systems, which is constantly developing in terms of treatment cost reduction.
- There is a clear trend of using microcomposite and nanocomposite materials in water-shutoff technologies to increase the strength of the water-shutoff gel screen; hydrodynamic modeling methods have also been actively used to increase the accuracy of the effect prediction and optimize the volume of injection of the water-shutoff composition.
- Self-diverting acid compositions based on viscoelastic surfactants are increasingly actively and successfully used in the well-acid stimulation technologies.
- The use of non-damaging block pills and killing fluids is becoming increasingly relevant due to the depletion of the fields being developed and the commissioning of deposits with hard-to-recover reserves. The use of gels based on polysaccharides (guar gum, xanthan gum, and modified cellulose) as remedial fluids allows for the production potential of wells during workovers to continue, minimizing the well-stabilization period.

## Figures and Tables

**Table 1:** Classification of gels used in the oil industry.

Application	Dispersion Medium	Dispersed Phase
Hydraulic fracturing	Water	1. Cross-linked polymer gels based on guar (cross-linkers: boron, titanium, and zirconium compounds). 2. Polacrylamide (slickwater, friction-reducing agent). 3. VES (cationic, zwitterionic, anionic surfactants, and their mixtures) and structure-forming agents (electrolytes, polymers, and nanoparticles).
	Hydrocarbons	Aluminum or iron alkyl phosphates
Water and gas shutoff	Water	1. PAM with Cr^3+^ or Al^3+^ cross-linkers. 2. PAM with organic cross-linkers. 3. Acidic and basic silicate gels. 4. Hybrid organic–inorganic gels. 5. Gels with microparticle and nanoparticle additives.
Conformance control and flow diversion	Water	1. PAM with Cr^3+^ or Al^3+^ cross-linkers. 2. Insoluble particles of cross-linked polymers (gel particle dispersions). 3. Cross-linked PAM with nanoparticles. 4. Aluminum oxychloride with modified PAM. 5. Sodium silicate with viscoelastic water-soluble cellulose derivatives.
EOR	Water	1. PAM with Cr^3+^ cross-linker. 2. Water-swellable phenolaldehyde resin. 3. PAM with a complex organic cross-linker—a mixture of formalin and resorcinol.
Stimulation with acidic compounds	Water	1. Hydrolyzed polyacrylonitrile and reagent based on inorganic gel. 2. VES
Well drilling	Water (WBM)	1. Cross-linked polymer-gel systems based on polyacrylamide–polyethylenimine, with organic cross-linkers (a mixture of resorcinol and paraform) reinforced with polypropylene fiber. 2. Water-soluble cellulose esters and modified starch combined with silicate reagents, lignosulfonates, calcium, potassium, and magnesium chloride salts, as well as caustic soda. 3. Hydrogels filled with mineral fibrous-dispersed materials. 4. Intercalated polymers.
Well killing	Water	1. Guar gum. 2. Xanthan gum. 3. Various types of cellulose (CMC, PAC, HEC). 4. Starch

## Data Availability

Not applicable.
